# Effect of telemedicine-supported structured exercise program in patients with chronic low back pain: a randomized controlled trial

**DOI:** 10.1371/journal.pone.0326218

**Published:** 2025-06-25

**Authors:** Yuan Feng, Chuanmei Zhu, Huizhen Liu, Tianjie Bao, Chongyang Wang, Zezhang Wang, Xiaoyi Wang, Ruishi Zhang, Yujia Zhang, Shaojun Zhang, Lin Yang, Siyi Zhu, Chengqi He

**Affiliations:** 1 Rehabilitation Medicine Center and Institute of Rehabilitation Medicine, Key Laboratory of Rehabilitation Medicine in Sichuan Province, West China Hospital, Sichuan University, Chengdu, China; 2 Department of Rehabilitation Medicine, The First Affiliated Hospital of Xi’an Jiaotong University, Xi’an, China; 3 Outpatient Department, West China Hospital, Sichuan University, Chengdu, China; 4 Center for Biostatistics, Design, Measurement and Evaluation (CBDME), West China Hospital, Sichuan University, Chengdu, China; 5 Department of Computer Science and Technology, Tsinghua University, Beijing, China; 6 Deyang Clinical Research Center for Rehabilitation Medicine, Mianzhu People’s Hospital, Mianzhu, Sichuan, China; Mugla Sitki Kocman Universitesi, TÜRKIYE

## Abstract

**Background:**

Structured exercise programs delivered via telemedicine have the potential to benefit patients with chronic low back pain (CLBP). However, evidence-practice gaps exist, such as low exercise adherence and lack of attention to the mental health of CLBP, so further research is needed to investigate the impact of telemedicine-supported structured exercise program on patients with CLBP.

**Objectives:**

To compare the clinical outcomes of patients with CLBP following a telemedicine intervention versus usual care therapy.

**Materials and methods:**

An open label, randomized controlled trial (RCT) was conducted over eight weekly sessions. Participants with CLBP were randomly assigned to either the Experimental Group (EG) or the Control Group (CG) in a 1:1 ratio, using a randomized numeric table. The EG received an intervention consisting of patient education, health coaching, and structured exercise program delivered through mobile health (mHealth) apps. The CG received usual care therapy, including patient education and paper handouts describing home exercises. The outcome measures included disability, pain intensity, mental health status, quality of life, walking ability, and exercise adherence. These were evaluated using the Roland Morris Disability Questionnaire (RMDQ), the Numerical Rating Scale (NRS), Depression-Anxiety-Stress Scale (DASS21), 12-item Short Form Health Survey (SF-12), Time Up and Go (TUG), and Exercise Adherence Rating Scale (EARS), respectively. Linear mixed-effects model analysis was conducted at baseline, after 4 weeks (during treatment), and after 8 weeks (post-treatment), adhering to the principles of intention-to-treat (ITT) analysis.

**Results:**

The study included a total of 78 participants, with 39 randomly assigned to each group. Changes were significantly different between groups at 8 weeks in disability (estimated value: −3.96, 95% CI: −5.45 to −2.47, P < 0.001), pain (estimated value: −1.69, 95% CI: −2.14 to −1.24, P < 0.001) and the physical health dimensions of quality of life (estimated value: 4.5, 95% CI: 1.29 to 7.71, P = 0.006). However, there were only within-group differences at 8 weeks in mental health status (estimated value: −3.81, 95% CI: −4.99 to −2.63, P < 0.001), mental health dimensions of quality of life (estimated value: 5.01, 95% CI: 2.9 to 7.13, P < 0.001), walking ability (estimated value: −0.92, 95% CI: −1.17 to −0.68, P < 0.001), and exercise adherence (Z: 1.91, P = 0.06) over time.

**Conclusion:**

This study suggests that a telemedicine-based structured exercise program is more effective than usual care therapy in improving disability, pain, and physical health dimensions of quality of life in patients with CLBP. Furthermore, the telemedicine program is equally effective as usual care therapy in enhancing mental health status, mental health dimensions of quality of life, walking ability, and exercise adherence. These findings indicate that implementing such a program could reduce the burden on patients with CLBP.

**Trial registration:**

This trial was registered at China Clinical Trial Registration Center with the identifier ChiCTR2300071560.

## Introduction

Low back pain (LBP) is the leading cause of disability worldwide, defined as pain located between the lower edge of the ribs and the gluteal crease, with or without leg pain. Approximately 80% of the population will experience LBP at least once in their lifetime. Of these, about 50% will recover within 2–3 weeks, while the remaining patients may progress to chronic low back pain (CLBP) [1]. CLBP is defined as LBP lasting more than three months [2]. CLBP is a significant contributor to annual reductions in quality of life due to disability, ranking sixth in the global burden of disease in both developed and developing countries [3,4].

Evidence increasingly suggests that low levels of physical activity in daily life are associated with increased pain, disability, and decreased quality of life in CLBP patients [5]. Exercise therapy has the potential to alleviate pain and enhance physical activity through both central and peripheral mechanisms. Research indicates that a well-designed exercise program can reduce pain by promoting cortical reorganization [6]. For the spinal system, exercise therapy improves spinal stability, leading to pain relief by enhancing muscle strength, endurance, and electrical activity [7]. Meta-analyses have shown that core stability training and motor control training are more effective than other treatments in reducing pain and disability in patients with CLBP [8,9]. However, in clinical practice, exercise therapy alone is often insufficient for the treatment of CLBP. Patients with CLBP tend to be more sedentary and have lower levels of physical activity than those without pain [10], often lacking the motivation to exercise. Additionally, pain-related fear and avoidance behaviors may lead to resistance to exercise, reducing adherence to therapy and limiting clinical outcomes [11,12]. Face-to-face health coaching by rehabilitation professionals can help overcome these barriers. Health coaching, based on behavioral change theory, encourages patients to adopt healthier lifestyles, develop sustainable habits, and improve exercise adherence [13,14]. Incorporating health coaching into structured exercise programs may therefore enhance outcomes. However, face-to-face exercise instruction is often time-consuming and costly (e.g., transportation, accommodation), and the recurrent nature of CLBP symptoms complicates long-term efficacy. Therefore, there is a need to explore a healthcare model that allows CLBP patients to exercise effectively at home.

Telemedicine has emerged as a promising solution, combining the benefits of health coaching and home exercise for chronic pain management [15]. Telemedicine offers advantages such as reduced travel, enhanced self-management, and increased time flexibility [16–18]. Patients can access health coaching from any location at any time, allowing them to tailor exercise programs to their specific needs. Evidence suggests that telemedicine positively impacts treatment adherence and reduces healthcare costs for various diseases [19]. Personalized content and real-time guidance through telemedicine, combined with structured exercise programs, have been shown to improve pain, disability, and overall quality of life compared to usual care [20]. However, a systematic review of 12 clinical trials found only low to moderate evidence supporting the effectiveness of telemedicine interventions in reducing pain intensity and disability in CLBP patients. There remains a lack of conclusive evidence on whether a structured telemedicine-based exercise program significantly improves symptoms in these patients. Furthermore, few studies have focused on the mental health aspects associated with CLBP, limiting the broader effectiveness of treatment. Additionally, current research lacks sufficient exploration of dynamic audiovisual monitoring and health coaching. Nicholl’s study, for example, investigated telemedicine-supported interventions for the self-management of CLBP and aimed to develop a mobile app offering tailored, algorithm-based digital interventions. However, this study was limited by its exclusive reliance on the app for managing patients’ exercise, without incorporating health coaching or videoconferencing [21]. This limitation highlights the need for further research into the effects of comprehensive telemedicine-based structured exercise programs on CLBP patients. To address this gap, our research incorporates dynamic videoconferencing monitoring and health coaching, potentially providing more robust evidence for the efficacy of telemedicine-based structured exercise programs in treating CLBP.

The objective of this study was to compare the clinical outcomes of CLBP patients who received a telemedicine-based structured exercise program with those who underwent usual care therapy. We hypothesized that at 8 weeks, the efficacy of the structured telemedicine-based exercise program in improving disability including pain would be better than that of the usual care group.

## Materials and methods

### Study design

This is a randomized controlled trial with two parallel arms, conducted in an open-label, difference-test manner, following a 1:1 allocation ratio, within a single-center over a span of 8 weeks. The trial follows the CONSORT guidelines (accessible in S3 File. CONSORT-2010-Checklist) [22]. The trial was prospectively approved by the Biomedical Ethics Committee of West China Hospital, Sichuan University (number 2022 Review (1976)) and registered on the Chinese Clinical Trial Registry (ChiCTR2300071560). Treatment occurred from May 15th, 2023, through May 10th, 2024. There are important changes to the study that will be reported to the Biomedical Ethics Committee of West China Hospital, Sichuan University for approval. Details of the Study Protocol have been shown elsewhere (accessible in S1 File. Study Protocol).

### Participants

Participants for the trial were recruited from Special Needs Outpatient Department at West China Hospital, Sichuan University, Wuhou District, Chengdu City, Sichuan Province, China. Those meeting the eligibility criteria were briefed on the trial procedure before being enrolled in the study after signing an informed consent form (accessible in S2 File). Inclusion and exclusion criteria were shown in Table 1. All study-related data was stored in an electronic data capture system. Experienced physical therapists independent of the trial design, intervention process, and statistical analysis accessed outcome measures at baseline, after the 4-week intervention, and after the 8-week intervention. Participants were considered dropouts if they: (1) abandoned the study; or (2) did not engage in any exercise session for 12 consecutive days in the Experimental group (EG) or missed 4 consecutive scheduled sessions in the Control group (CG) [23,24].

**Table 1:** Inclusion and exclusion criteria.

Inclusion criteria	Exclusion criteria
All applicants must be between 18 and 65 years old.	Suffering from a specific disease of the spine, such as infection, spinal tumor, spinal tuberculosis, fracture, spondylolisthesis, isthmus, or aneurysm.
Pain, and muscle stiffness are located below the coastal margin and above the gluteal fissure fold, with or without lower limb pain [1].	Pain caused by other diseases.
Pain lasts 12 weeks or more.	Cognitive impairment results in the inability to understand the physical therapist’s instructions and the content of the app.
Pain intensity (when the worst pain) ≥ 3 on the Numeric Rating Scale (NRS).	Pregnant or breastfeeding.
Ability to operate a smartphone.	History of spine surgery.
Voluntarily participate in the trial and sign the informed consent.	Patients who have received exercise therapy in the past three months for low back pain.
Accepted randomization.	Patients with severe cardiovascular and cerebrovascular diseases.
Candidates who meet all the above criteria will be included	Candidates meeting any of the above criteria will be excluded

### Randomization and blinding

#### Sequence generation.

Randomization was carried out as participants are recruited using block randomization with a computer-generated random sequence. We chose 4 as the block. A chosen data manager had been tasked with creating random sequences and safely storing them in IBM SPSS, version 26.0.1. This manager was not involved in the recruitment, intervention, or evaluation processes.

In addition, the randomization list was safely kept in a secured, locked area of our building as well as online in a password-protected database. Throughout the duration of the project, the randomization list was only accessible to authorized people, such as the primary investigator and designated data manager, guaranteeing its integrity. This configuration not only protected the list but also enabled an organized procedure that is not impacted by individual staff changes.

#### Concealment mechanism.

The assignment codes were inserted into sequentially numbered, sealed, opaque envelopes by an independent researcher who is not participating in the experiment, in accordance with the computer-generated random sequence findings. This obscured group allocation. According to the experimental design, participants were assigned to the “control group” or “experimental group” based on the matching “0” or “1” on the random number card, once it has been opened in accordance with the prescribed protocols.

#### Implementation.

In the enrollment stage, a physician who is not involved in the randomization procedure assessed individuals. After this assessment, the assignment group was determined on-site by opening sealed and opaque envelopes, and participants then were divided into the appropriate groups at a 1:1 ratio. Permuted blocks of size 4 was used at random to guarantee a fair distribution among the groups.

#### Blinding.

It is not possible for blind doctors and volunteers to do group assignments. Since the subjects are not blinded and the results are self-reported, it is decided not to blind the assessors. While there is no blinding of participants, physicians, or assessors, other techniques are used to reduce bias. To minimize the possibility of bias resulting from unblinding, the trial hypothesis is specifically kept secret from both assessors and participants.

### Interventions

The physiotherapists who delivered the intervention in both two groups were not involved in the outcome measures and were not blind to the group assignment. The two groups conducted an 8-week intervention, for a total of 24 sessions. The interventions in both groups were completed over 8 weeks, aiming to observe a short-term effect, following the 2020 clinical practice guidelines of the North American Spine Association [25]. Subjects were encouraged to participate in the intervention and assessment throughout the trial protocol. Frequency of attendance, medication changes, adverse events, etc. were reported by the participants to the physiotherapist in charge of the trial management and were duly recorded in the case report form.

#### EG therapy.

The intervention measures for the experimental group included app-based exercise therapy (40 minutes per session, 3 times per week, for 8 weeks), patient education (10 minutes per session, 1 time per week, for 8 weeks), and WeChat video-based health coaching (20 minutes per session, 1 time per week, for 8 weeks).

##### App-based exercise therapy

At the initial visit, the physician implemented the app-based exercise therapy in three parts: guiding the subject through login of the “Shu Kang PRO” app, formulating an individualized exercise prescription, demonstrating the app-based exercises, and providing guidance to the therapist. S1 Fig displays details of the three parts, while S2 Fig shows the specific movements taught by the therapist only involved in the intervention process. Exercise prescriptions were developed using baseline data and FITT-VP principles, consisting primarily of core stability and motor control training as recommended by clinical guidelines [25]. The doctor tailored the stepwise treatment plan to adjust for individual variability.

Patients could schedule exercise time according to their own schedule through the alarm in the app. Physicians can set phased goals for subjects in advance, and regularly remind patients through the app whether the goals are completed or not. After being taught all the movements during the initial consultation, all participants underwent an eight-week training program. The program consisted of 3 sessions per week, each lasting 40–60 minutes, under the supervision of a physical therapist. Each session included a warm-up, functional training, and relaxation.

##### App-based patient education

An animated science movie about CLBP was shown to the patient by the physical therapist during the baseline assessment. Patients received eight instructional illustrated pieces about CLBP via the app once a week. It took five to ten minutes to read each article [26]. It covers the following topics: definition, pathogenesis, etiology, diagnosis, treatment, management of day-to-day activities, prevention of the progression and recurrence of CLBP, diet and weight control, etc. Patients received a self-administered CLBP knowledge questionnaire via the app every Friday. The purpose of the questionnaire findings is to determine whether patients have read and comprehended the CLBP information provided through the app, not for statistical analysis. Patients are allowed to respond to the questionnaire more than once until they choose the right responses.

##### WeChat video-based health coaching

A group WeChat video was held once a week (each video lasts 40 minutes), which means 8 out of 24 sessions are supervised by therapists. At the same time, the physical therapist could communicate with the patient, including the progress of the disease, diet, weight, and other topics, and the patients could also communicate with each other. Health coaching based on group video can enable patients to provide peer assistance and psychological support, improve the adhesion between therapists and patients, and increase the sense of trust between them. In addition, a weekly meeting can be used to check that the patient’s movements are accurate so as not to cause new injuries. If the patient’s movements are accurate and the progress indicators are met, the therapist can also make a judgment through the meeting to advance the patient’s treatment plan.

#### CG therapy.

The control group’s interventions included paper-based exercise therapy(40 minutes per session, 3 times per week, for 8 weeks) and app-based patient education (10 minutes per session, 1 time per week, for 8 weeks, same as EG, but unable to access the exercise therapy section in the app).

##### Paper-based exercise therapy

At the initial diagnosis, physical therapists involved only in the intervention process distributed a printed exercise manual to patients. The manual contains the same training movements and frequency as the experimental group. Therapists taught the training maneuvers, with no further teaching or instruction during the subsequent 8-week course of treatment unless the patient requests help. The exercise manual marks the essentials, standards, and precautions of each movement in the form of graphic and text combinations. If patients have problems exercising at home, they can seek the help of the therapist at the nearest designated partner community hospital or first visit the hospital. Physical therapists at designated community hospitals are trained in advance. A total of 24 unsupervised self-training sessions were conducted 3 times a week for 8 weeks.

### Relevant concomitant care permitted or prohibited during the trial

All participants were provided with the same version of the app, which remained unchanged throughout the trial. During the treatment phase, if a participant’s condition deteriorates, they are permitted to utilize any relevant treatment to manage the disease, such as drug therapy or physical therapy. However, engaging in additional exercise therapy is prohibited. Participants are required to report any such additional treatments accurately to the investigator, who documented them in the case report forms.

### Outcome measures

Outcomes were collected at baseline, 4, and 8 weeks (except exercise adherence at 4 and 8 weeks). The changes of primary (disability including pain) and secondary (mental, quality of life, walking, and adherence) outcomes were calculated between baseline and 8 weeks.

#### Disability.

Disability was evaluated using the Roland Morris Disability Questionnaire (RMDQ), including 24 items specifically affected by low back pain, each question is limited by the phrase “because of my back pain” to distinguish it from other causes of dysfunction, thus making it easier for the patient to answer and avoiding confusion [27]. The score for each question is 1 point, 1 point for answering “yes”, and 0 points otherwise, the highest total score is 24 points, and the lowest is 0 points. The higher the score, the higher the degree of dysfunction.

#### Pain intensity.

Pain was evaluated using the Numerical Rating Scale (NRS), which is composed of 11 numbers from 0 to 10. The patient uses 11 numbers from 0 to 10 to describe the degree of pain. The larger the number, the more severe the pain [28].

#### Mental health status.

Mental health status was measured by Depression-Anxiety-Stress Scale (DASS21), which is a scale for assessing mental health based on a three-factor model of depression-anxiety-stress. This scale uses the degree of various induced negative emotional states of the subjects as an evaluation index and adopts a 4-point scale scoring method, and higher scores indicate higher depression, anxiety, and stress indices [29].

#### Quality of life.

Quality of life was measured by 12-item Short Form Health Survey (SF-12), which is a simplified version of the universal and concise quality of life scale SF-36 developed by the Boston Institute of Health Education. The scale has 12 items and evaluates 8 dimensions of health-related quality of life [30].

#### Walking ability.

Walking ability was measured by Time Up and Go (TUG), which is a rapid assessment of walking function. Record the time it takes from standing to folding back three meters and sitting down [31,32].

#### Exercise adherence.

Exercise adherence was measured by Exercise Adherence Rating Scale (EARS), which is a 16-item scale developed to evaluate the adherence of individuals to the exercises recommended for individuals with chronic pain diseases, and the reasons for their compliance or non-compliance [33].

### Safety and adverse events

Subjects in the EG group were supervised by weekly videoconferences to reduce the risk of injury during exercise, while subjects in the CG group were able to go to the partner community hospital for help in case of adverse reactions or doubts during exercise.

### Sample size calculation

The sample size is calculated by G*power 3.1.9 based on the following conditions. According to the standard of Cohen’s effect size, 0.2, 0.5, and 0.8 are the boundary values of small, medium, and large effect sizes respectively [34]. Select 0.3 to verify the small to medium effect size, based on Murtezani ‘s test results [35], α is 0.05, power is 0.8, and the correlation amongst repeated measures is 0.5. The final calculated sample size was 58. Considering the 25% dropout rate, the actual required sample size should be 78.

### Data collection and management

#### Plans for assessment and collection of outcomes.

Data were collected through both paper survey and online. If the subject can come to the hospital for follow-up, the paper survey will be used, otherwise the online method will be used. In case of paper survey, the assessor printed and completed all examination forms during each examination; to provide better uniformity, the assessor got uniform training. The patients also printed out the questionnaires and filled them out. In case of online collection, the scale was made into an electronic questionnaire to be sent through an app and the TUG test was done through video conferencing. This information was entered by two data administrators into anonymous databases that are set up with logical glitches such as required fields and response limits that defy common sense. The information in the tables then were used for statistical analysis.

#### Data management.

The outcome assessors recorded all experimental procedures and data in a case report form. To protect patient privacy, a special identifying code was given to each participant. Only the outcome assessors and the corresponding author had access to the case report forms. Each data entry was verified twice by two separate and impartial assessors. The information entered and validated on the case report forms become non-editable.

### Statistical analysis

Baseline outcome measurements, group comparability, and descriptive characteristics were thoroughly assessed. Given the potential for patient loss and dropout during the study and follow-up, it was crucial to include both intention-to-treat (ITT) and per-protocol (PP) analyses. PP analysis examines data only from participants who completed the entire trial after randomization, while ITT analysis incorporates follow-up data from all randomized participants [36]. While PP analysis may exaggerate differences between groups, ITT analysis helps to mitigate bias from protocol deviations, reduces the risk of follow-up loss, and generally produces more conservative and less pronounced differences. To ensure accurate assessment of effectiveness, ITT analysis was chosen as the primary analysis set for this study.

Statistical analyses were conducted using IBM SPSS, version 26.0.1, with a significance threshold set at P < 0.05 for two-sided tests. Categorical variables (e.g., sex, occupation, education level) were reported as numbers (n) and percentages (%), while continuous variables (e.g., age, BMI, pain intensity, duration of low back pain) were expressed as mean ± standard deviation for normally distributed data or median with interquartile range (quartile 1-quartile 3) for skewed distributions. Comparisons between the experimental and control groups were performed using independent samples T-tests for normally distributed continuous variables, Mann-Whitney U tests for skewed continuous variables, and chi-square tests for categorical variables. Additionally, if the trial results meet the assumptions of homogeneity of variance and sphericity, a two-factor, three-level repeated measures analysis of variance (group*time) was applied; otherwise, a mixed-effects model was utilized.

### Consent for publication

Written informed consent were obtained from the patient for publication of this RCT and any accompanying images. A copy of the written consent is available for review by the Editor-in-Chief of this journal.

## Results

### Participant characteristics

**Fig 1:**
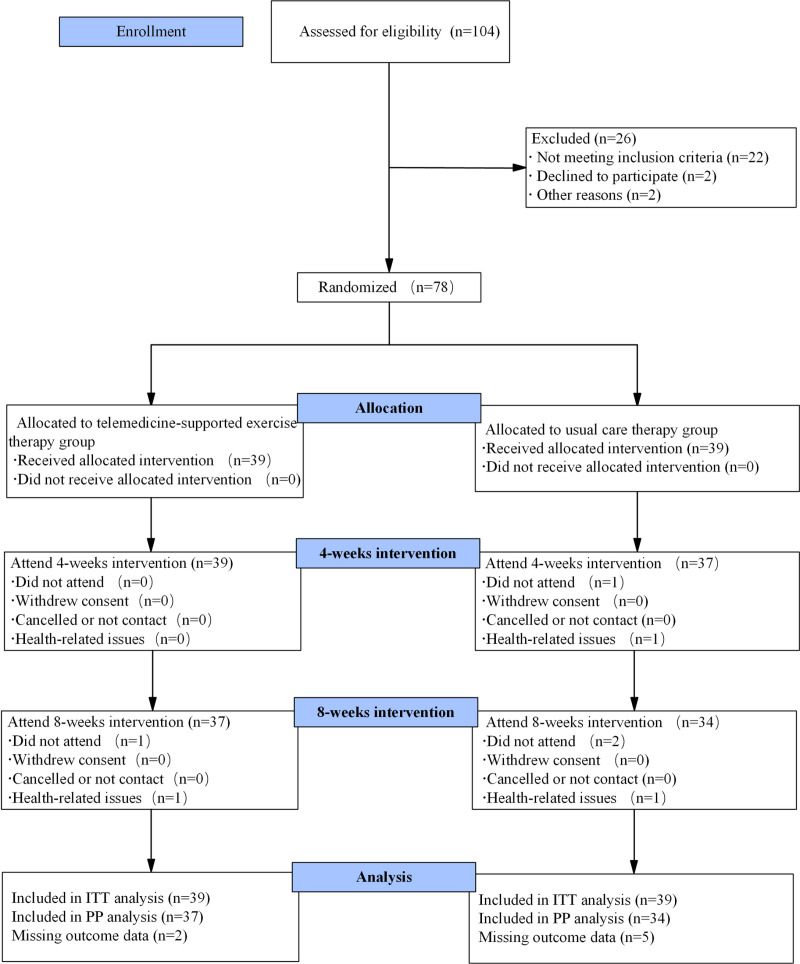
Study flow diagram.

Eligibility screening was conducted for 104 participants, resulting in 22 ineligible, 2 who declined participation, and 2 exclusions. A total of 78 participants were randomly assigned to either the telemedicine-based exercise therapy group (EG) or the usual care therapy group (CG), with 39 participants in each group. The completion rate was 94.9% (37/39) in the EG and 87.2% (34/39) in the CG (Fig 1).

The demographic characteristics of the patients included in this study are presented in Table 2. The analysis revealed that there were no statistically significant differences between the two groups in terms of age, gender, or other demographic variables (P > 0.05).

**Table 2:** Baseline characteristics of study participants. (N = 78).

Characteristic		EG (n = 39)	CG (n = 39)	*P*
Age(years), M (QU-QL)		27(21-34)	22(21-37)	0.47^a^
Height(cm), x̄ ± SD		170 ± 10.37	168.21 ± 8.38	0.13^b^
Weight(kg), M (QU-QL)		62(55-73)	58(52.5-71)	0.46^a^
BMI (kg/m^2^), M (QU-QL)		22 (20.5-23.6)	22(19.9-23.9)	0.73^a^
Duration of low back pain(months), M (QU-QL)		24(6-84)	30(18-48)	0.38^a^
Gender, n (%)				0.26^c^
	Man	22(56.4)	17(43.6)	
	Woman	17(43.6)	22(56.4)	
History of low back pain, n (%)				0.53^c^
	Yes	32(82.1)	34(87.2)	
	No	7(17.9)	5(12.8)	
Pain intensity, n (%)	Mild	16(41)	20(51.3)	
	Moderate	20(51.3)	17(43.6)	
	Severe	3(7.7)	2(5.1)	
Educational level, n (%)				0.12^c^
	Junior high school	3(7.7)	1(2.6)	
	High School	3(7.7)	0(0)	
	College	0(0)	3(7.7)	
	Undergraduate	24(61.5)	30(76.9)	
	Master’s Degree	7(17.9)	4(10.3)	
	PhD	2(5.1)	1(2.6)	
Taking painkillers, n (%)				0.47^c^
	Yes	14(35.9)	11(28.2)	
	No	25(64.1)	28(71.8)	
Recruitment Channels, n (%)				0.23^c^
	Outpatient	15(38.5)	10(25.6)	
	Other	24(61.5)	29(74.4)	
With hip and leg symptoms, n (%)				0.50^c^
	With	22(56.4)	19(48.7)	
	Without	17(43.6)	20(51.3)	
Exercise regime, n (%)				0.11^c^
	Yes	19(48.7)	26(66.7)	
	No	20(51.3)	13(33.3)	
Smoking history, n (%)				1^c^
	Yes	5(12.8)	4(10.3)	
	No	34(87.2)	35(89.7)	
Drinking history, n (%)				0.50^c^
	Yes	4(10.3)	6(15.4)	
	No	35(89.7)	33(84.6)	

Abbreviations: BMI, Body Mass Index.

^a^Mann-Whitney U test.

^b^Two independent samples T-test.

^c^Chi-square test.

### Overview of clinical outcomes

**Fig 2:**
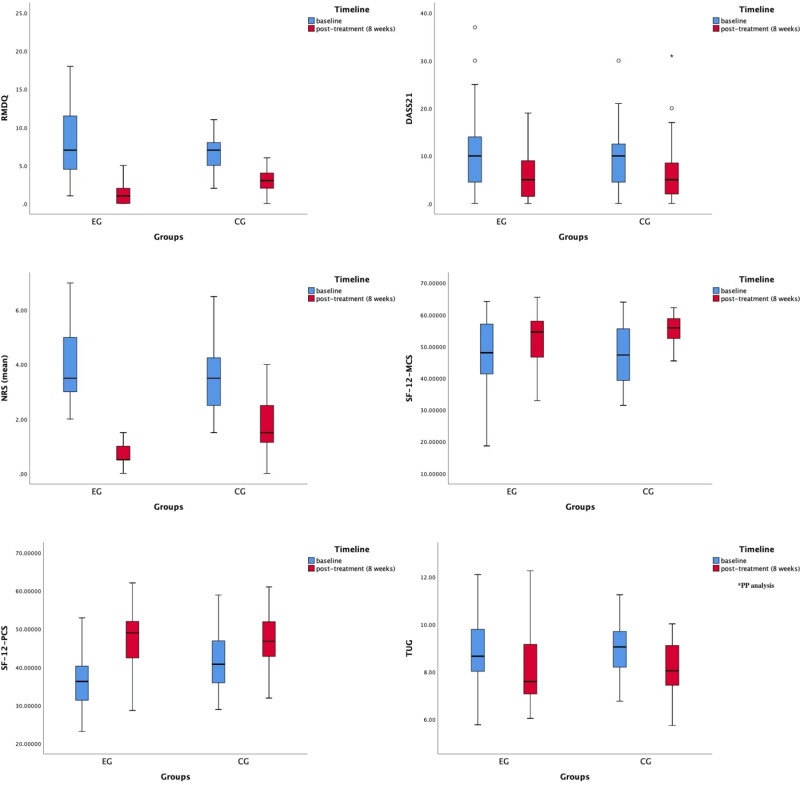
Outcomes changes of box plots (except for the exercise adherence indicator): ITT analysis.

The results showed no statistically significant differences between the two groups in all clinical outcome index scores before treatment (P > 0.05), confirming their comparability. Post-treatment, all outcome indicators improved in both groups compared to pre-treatment. Table 3 presents the interaction effects observed in the ITT analysis for each indicator, except for the exercise adherence indicator. Table 4 and Fig 2 display the ITT analysis results using the mixed linear effects model for all clinical outcome indicators except for adherence, while the per-protocol analysis results are provided in S1 and S2 Tables, and S3 Fig.

**Table 3:** Interaction effect test for each indicator (except for the exercise adherence indicator). (N = 78).

Outcome Variables		Cases	P-value for between-group effect (Group)	P-value for within-group effect (Time)	P-value for interaction effect^#^ (Group^*^time)
RMDQ		234	**0.04**	**<0.001**	**<0.001**
NRS (mean value)		234	**0.002**	**<0.001**	**<0.001**
DASS21		234	0.63	**<0.001**	0.36
SF-12					
	PCS	234	0.22	**<0.001**	**0.02**
	MCS	234	0.83	**<0.001**	0.74
TUG		178	0.84	**<0.001**	0.97*

Abbreviations: PCS, physical component summary; MCS, mental component summary.

# Linear mixed-effects model for the repeated-measures analysis using the compound symmetric covariance structure.

* Linear mixed-effects model based on PP analysis.

**Table 4:** Outcomes changes (except for the exercise adherence indicator) * ITT analysis. (N = 78).

Outcome Variables (median/mean)	N	EG	N	CG	Estimate value	P^#^
Comparison between groups						
**RMDQ**	39		39			
Baseline		7(4-12)		7(5-8)	−3.96	**<**0.001
8 weeks		1(0-2)		3(2-4.25)		
**NRS**	39		39			
Baseline		3.5(3-5)		3.47 ± 1.28	−1.69	**<**0.001
8 weeks		0.5(0.25-1)		1.96 ± 1.03		
**SF-12-** **PCS**	39		39			
Baseline		37.1 ± 7.18		41.51 ± 7.41	4.5	0.006
8 weeks		46.85 ± 8.02		46.80 ± 7.81		
Comparison within group						
**DASS21**	39		39			
Baseline		10(4-15)		10(4-13)	−3.81	**<**0.001
8 weeks		5(1-10)		4(2-9)		
**SF-12-** **MCS**	39		39			
Baseline		47.78 ± 10.31		47.18 ± 9.14	5.01	**<**0.001
8 weeks		54.77(45.9-58.45)		56.51(49.17-59.01)		
**TUG**	10		12			
Baseline		8.9 ± 1.46		9 ± 1.12	−0.92	**<**0.001^*^
8 weeks		7.6(7.07-9.2)		8.05 ± 1.13		

# Linear mixed-effects model for the repeated-measures analysis using the compound symmetric covariance structure.

* Linear mixed-effects model based on PP analysis.

### Impact on disability

For RMDQ scores, the linear mixed model analysis revealed an interaction effect, and further analysis of between-group effects showed significant differences between the two groups (−3.96, 95% CI: −5.45 to −2.47, P < .001; Table 4).

### Impact on pain intensity

For NRS scores, the linear mixed model analysis identified an interaction effect, and further analysis of between-group effects indicated significant differences between the two groups (−1.69, 95% CI: −2.14 to −1.24, P < .001; Table 4).

### Impact on mental health status

For DASS21 scores, the linear mixed model analysis did not reveal an interaction effect. A one-effect analysis of time effects was therefore performed, showing statistically significant within-group comparisons (−3.81, 95% CI −4.99 to −2.63, P < .001; Table 4), but no significant between-group differences.

### Impact on quality of life

Quality of life encompasses both physiological and psychological dimensions, which we measured using the SF-12 questionnaire, which consists of the SF-12-PCS and SF-12-MCS dimensions. After linear mixed model analysis, an interaction effect was found for SF-12-PCS, and further analysis of the between-group effect revealed a significant difference between the two groups (4.5, 95% CI 1.29 to 7.71, P = 0.006; Table 4). For SF-12-MCS, no interaction effect was observed, so only a time effect analysis was performed. The within-group comparisons were statistically significant (5.01, 95% CI 2.9 to 7.13, P < .001; Table 4), with no between-group effect.

### Impact on walking ability

The TUG indicator required patients to visit the hospital multiple times for offline measurements or through online video, resulting in lower acceptability and compliance. Consequently, 16 cases were lost after 4 weeks of intervention, and 28 cases were lost after 8 weeks. Due to the substantial number of missing values, blind interpolation could lead to inaccurate results. Therefore, missing values for the TUG indicator were not interpolated, and only PP analyses using actual data were conducted to report the results.

For TUG scores, the linear mixed model analysis did not reveal an interaction effect. A one-effect analysis of time effects showed statistically significant within-group comparisons (−0.92, 95% CI −1.17 to −0.68, P < .001; Table 4), but no significant between-group differences.

### Impact on exercise adherence

For the exercise adherence indicator, a nonparametric rank-sum test was used for statistical analysis since adherence could not be measured at baseline, precluding repeated measures, and the data did not follow a normal distribution. The nonparametric rank-sum test results indicated no statistically significant differences between the two groups’ EARS scores at 4 and 8 weeks of intervention (P > 0.05), suggesting that the intervention did not affect EARS scores between the groups. Table 5 presents the results of the EARS index analysis using the nonparametric rank-sum test (PP analysis is provided in S3 Table).

**Table 5:** Comparison of EARS at each time point between the two groups *ITT analysis. (N = 78).

	T1	T2	Difference
EG	49(46~51)	48(45~56)	0(−2~3)
CG	43.68 ± 3.5	45.35(43~48)	2(0~3)
Z	−5.5	−3.19	1.91
P^*^	**<**0.001	0.001	0.06

Abbreviations: T1, Last day at 4 weeks of intervention; T2, last day at 8 weeks of intervention.

* Mann-Whiteney U test.

## Discussion

In this study, significant improvements were observed in both groups regarding disability, pain intensity, mental health status, quality of life, and walking ability before and after the intervention. Statistically significant differences between the groups were found in disability, pain, and the physical health dimensions of quality of life. However, no significant differences were noted in mental health status, the mental health dimensions of quality of life, and walking ability between the groups. Both groups demonstrated high adherence to the program, with similar treatment doses administered. Although the telemedicine-supported exercise therapy group had a lower dropout rate, no statistically significant difference in adherence was found between the two groups. The results confirmed the additional contribution of telemedicine structured exercise program in disability, pain, mental health status, quality of life and walking ability. Moreover, after 8 weeks of intervention, both groups showed an improvement of more than 2 points in the NRS to reach the minimal clinically significant difference (MCID) [37], thus having clinical significance. Since the importance of continuity in long-term rehabilitation in patients with CLBP is comprehended, telemedicine structured exercise program could be considered to be promoted in clinical applications.

Telemedicine was used in this study to provide movement and postural corrections via audio-visual feedback, which contributed to pain reduction and a decreased risk of sports injuries. This intervention improved spinal flexibility and function, leading to greater reductions in disability and pain intensity among CLBP patients compared to the control group. However, mental health status is influenced by more complex factors [38,39], such as work and family factors, which may explain the lack of significant differences in mental health outcomes between the two groups. Despite this, both groups showed improvements from baseline to post-treatment, indicating that telemedicine is a viable alternative to usual care therapy.

Severe LBP and associated symptoms in the hips and legs can significantly impair walking ability in CLBP patients. Improvements in walking ability reflect symptom amelioration and provide an objective measure of patient progress. In this study, the TUG test, a widely accepted and accessible method, was used to assess walking ability [31]. Both intervention groups showed reduced TUG times after 8 weeks of treatment, but no statistically significant difference was observed between the groups. This lack of differentiation may be due to the mild severity of LBP among the participants, which may have minimized the impact on their walking ability. Consequently, despite the observed improvements, the limited scope for further enhancement made it difficult to detect statistically significant differences between the groups.

The involvement of geographically remote patients in this remote rehabilitation study posed challenges in collecting objective indicators. Despite the advantages and disadvantages of this approach, the study aimed to broaden its participant base by including interested patients. Four patients from the outpatient clinic expressed a desire to participate but faced difficulties returning home after their consultations. As a result, informed consent and baseline data were collected online. However, due to site constraints, TUG measurements were not performed, leading to baseline TUG data for only 74 patients. By week 4, 16 patients were lost to follow-up—10 due to significant symptom improvement and 6 due to scheduling conflicts. By week 8, an additional 28 patients were lost—16 due to symptom improvement and 12 due to time conflicts that precluded TUG testing. Although the loss of subjects affected this indicator, real-world research highlights the challenges of measuring objective indicators in telemedicine. Future studies should consider integrating wearable devices or augmented reality (AR) technology to collect data from patients’ at-home exercises, enabling an objective evaluation of the efficacy of telemedicine-supported exercise therapy for CLBP patients.

Adherence to telemedicine has historically been challenging [40]. Previous studies have shown that telemedicine has similar or lower dropout rates compared to usual care therapy [41,42]. In this study, both groups performed exercises at home. The experimental group had a lower dropout rate (5%) compared to the control group (18%). This difference may be due to the dynamic video support and weekly wellness coaching provided by the physical therapist, which likely enhanced the enjoyment of the exercises and fostered greater patient-physician engagement [43,44]. Home exercise adherence was assessed using the standardized EARS [33]. The findings revealed no statistically significant difference between the two groups, suggesting that the intervening factors did not significantly influence EARS scores. The absence of significant between-group differences may be due to the study’s insufficient sample size. Increasing the sample size or extending the duration of treatment could potentially reveal between-group variability, a limitation that should be addressed in future studies. Additionally, improving information exchange could further enhance exercise adherence in the experimental group by increasing patients’ perception of being supported.

There have been many studies confirming the effectiveness of tele-rehabilitation in the treatment of CLBP, such as a double-blind, two-armed randomized controlled trial by Fatih Özden on 50 patients with CLBP, where participants were randomly assigned to either the tele-rehabilitation group or the conventional rehabilitation group [45]. After 8 weeks of treatment, the tele-rehabilitation group achieved significant improvements in pain, function, quality of life, kinesiophobia, satisfaction and motivation (p < 0.05). In addition, the telerehabilitation group reported a more significant increase in all parameters compared to the conventional rehabilitation group. This is consistent with the findings of our study, however, the intervention in their study consisted of exercise therapy only and lacked patient education as well as physiotherapist-based health coaching and real time feedback, which was remedied in our study, thus both groups in our study showed statistically significant improvement in disability and pain before and after treatment, and the experimental group was superior to the control group.

However, two other studies by Fanuscu, Aybüke, and Weihong Shi concluded that the tele-rehabilitation group was comparable to the clinical rehabilitation group [46,47], and we consider the reason for this to be that both researchers used face-to-face exercise therapy in the control group, which yielded better results than the home-based paper version of exercise therapy used in the control group in our study. Therefore, their study concluded that the two groups were comparable, while the experimental group in our study performed better than the control group in terms of disability and pain.

The study’s strength lies in its ability to engage patients in treatment and self-management. Smartphone apps can cost-effectively provide education and encourage healthy behaviors, while WeChat video-based health coaching can offer exercise guidance and monitor participation [48]. However, several design limitations exist. First, the requirement for smartphone use may have limited participation to individuals with lower socioeconomic status and education levels, introducing sample selection bias and limiting the generalizability of the findings. Second, the nature of the intervention made it difficult to blind both patients and therapists. To mitigate potential bias from unblinding, the trial implemented strict confidentiality measures for outcome assessors and patients.

Further investigation is needed to determine the most effective strategies for telemedicine implementation, particularly in measuring objective outcomes and improving adherence to prescribed exercises. Additionally, studies with long-term follow-up and cost-effectiveness analyses are crucial. Developing more suitable healthcare models for patients with chronic pain must prioritize patient convenience without compromising long-term efficacy or cost-effectiveness.

## Conclusion

This study indicates that a structured telemedicine-based exercise program enhances recovery in CLBP patients to a greater extent than usual care therapy in terms of disability, pain, and the physical health dimensions of quality of life. Additionally, it promotes recovery equivalent to usual care therapy concerning mental health status, the mental health dimensions of quality of life, walking ability, and exercise adherence. This approach holds promise as a potential strategy to alleviate the burden on CLBP patients.

## Supporting information

S1 File Study Protocol. (PDF)

S2 File Informed consent form. (PDF)

S3 File CONSORT-2010-Checklist. (DOC)

S1 Fig Flowchart of medical consultation. (TIF)

S2 Fig Specific movements. (TIF)

S3 Fig Outcomes changes of box plots (except for the exercise adherence indicator): PP analysis. (TIF)

S1 Table Interaction effect test for each indicator (except for the exercise adherence indicator) * PP analysis. (DOCX)

S2 Table Outcomes changes (except for the exercise adherence indicator) * PP analysis. (DOCX)

S3 Table Comparison of EARS at each time point between the two groups *PP analysis. (DOCX)
