# Supplementary material for: Effect of telemedicine-supported structured exercise program in patients with chronic low back pain: a randomized controlled trial
Source: PLoS One. 2025 Jun 25;20(6):e0326218. doi: 10.1371/journal.pone.0326218 (PMC12193851; doi:10.1371/journal.pone.0326218)
Supplement: S1 File — (PDF) [file pone.0326218.s001.pdf]

[Study protocol]

[Yuan Feng] et al

Effect of telemedicine-supported structured exercise program in patients with chronic low back pain: study protocol for a randomized controlled trial

Yuan Feng[1, 2, 3]*, Chengsen Jia[1, 2]*, Huizhen Liu[4], Tianjie Bao[1, 2], Chongyang Wang[1, 2, 5], Zezhang Wang[1, 2,] Jielei Huang [1, 2], Yiwei Jiang [1, 2], Xiaoyi Wang[1, 2], Ruishi Zhang[1, 2], Yujia Zhang[1, 2], Shaojun Zhang[6], Siyi Zhu[1, 2], Chengqi He[1, 2]

[1]Rehabilitation Medicine Center and Institute of Rehabilitation Medicine, West China Hospital, Sichuan University, Chengdu, China; [2]Key Laboratory of Rehabilitation Medicine in Sichuan Province, West China Hospital, Sichuan University, Chengdu, China; [3]Department of Rehabilitation Medicine, The First Affiliated Hospital of Xi'an Jiaotong University, Xi'an, China; [4]Center for Biostatistics, Design, Measurement and Evaluation (CBDME), West China Hospital, Sichuan University, Chengdu, China; [5]Department of Computer Science and Technology, Tsinghua University, Beijing, China; [6]Department of Rehabilitation Medicine, Deyang Clinical Research Center for Rehabilitation Medicine, Mianzhu People's Hospital, Mianzhu, China.

Yuan Feng[1, 2, 3]*;

Chengsen Jia[1, 2]*

*These authors contributed equally to this work

Correspondence: [Siyi Zhu]

[Department of Rehabilitation Medicine, West China Hospital, Sichuan University, #37 Guoxue Alley, Wuhou District, Chengdu, Sichuan, PR China.]

Information Classification: General

Email (hxkfzsy@scu.edu.cn)

Abstract:

Purpose: Telemedicine-based structured exercise programs have the potential to benefit patients with chronic low back pain (CLBP). However, evidence-practice gaps persist, including low exercise adherence and insufficient focus on mental health. Consequently, further research is warranted to clarify the impact of telemedicine-supported structured exercise programs on patients with CLBP.

Patients and methods: This randomized controlled trial will include patients with CLBP who have not received exercise therapy in the past three months. Participants in the trial group will receive patient education, health coaching, and home-based exercise therapy via mobile health (mHealth) applications loaded with wearable device integration. Those in the control group will receive conventional treatment, comprising patient education and written instructions for home exercises. Disability including pain intensity will be assessed as primary outcomes using the Roland-Morris Disability Questionnaire (RMDQ) and the Numerical Rating Scale (NRS) at baseline, 4 weeks, and 8 weeks. Statistical analyses will depend on assumptions such as homogeneity of variance and sphericity: if met, a two-factor, three-level repeated measures analysis of variance will be used; otherwise, a linear mixed-effects model will be employed.

Keywords: low back pain; telemedicine; exercise; study protocol; randomized controlled trial.

Introduction

Globally, low back pain (LBP) is the leading cause of disability, defined as pain located between the lower costal margin and the upper gluteal fold, with or without leg pain. [1] Chronic low back pain (CLBP) refers to LBP lasting more than three months. Approximately 80% of individuals experience at least one episode of LBP in their lifetime, with about half recovering within 2–3 weeks and the remainder gradually developing CLBP. [2] CLBP is a major contributor to disability in both developed and developing countries, ranking sixth in overall disease burden. [3,4] In China, the annual prevalence of adult LBP ranges from 20.88% to 29.88%, underscoring its

Information Classification: General

significance as a public health issue. [5] As a global public health concern, CLBP significantly disrupts daily life and work, causing pain, limited function, anxiety, depression, and reduced working hours.

Growing evidence indicates that baseline anxiety, depression, and low levels of physical activity correlate with increased pain, greater disability, and reduced quality of life in CLBP. [6] Exercise therapy can alleviate pain through both central and peripheral mechanisms, including promoting cortical reorganization [7] and modulating pro- and anti-inflammatory cytokines. [8,9] By enhancing muscle strength, endurance, and electrical activity, exercise also improves spinal stability and relieves pain. [10] Several meta-analyses have shown core stability and motor control training to be more effective than other therapies in reducing pain and disability among CLBP patients. [11,12] However, exercise therapy alone is often insufficient because CLBP patients tend to engage in sedentary behaviors and may lack motivation, leading to fear avoidance and suboptimal adherence. [13-15] Poor adherence in CLBP patients may make exercise therapy less effective, leading to concealment of the true efficacy. Unsupervised exercise further raises risks of injury, [16] and although it can mitigate pain and disability, it often suffers from poor compliance. [17] Semi-structured interviews reveal that younger patients favor visual or dynamic support, while older patients prefer coaching and supervision. [18] Health coaching, grounded in behavioral change theory, promotes healthier lifestyles and can improve self-management and adherence. [19,20] Nevertheless, in-person supervision or coaching is time-intensive and costly, [21] and long-term efficacy may be jeopardized by recurrent symptoms when patients must continue exercising at home.

To optimize home exercise for CLBP, telemedicine—which harnesses the Internet, mobile devices, and other digital platforms—has shown strong potential for prevention, treatment, and health promotion. [22] Research indicates that telemedicine enhances treatment adherence and lowers healthcare costs for various conditions. [23] When structured exercise programs are delivered via telemedicine, patients can conveniently access personalized information and real-time guidance, improving pain, physical function, and overall quality of life compared with usual care. [24,25] Although a systematic review of 12 clinical trials suggests low to moderate evidence for

Information Classification: General

telemedicine's effectiveness in alleviating pain intensity and improving function in CLBP, challenges persist in achieving high exercise adherence, adequately addressing mental health factors, and incorporating objective outcome metrics. [26]

In response to these gaps, we will conduct a randomized controlled trial using a wearable-device–integrated app to remotely monitor home exercises for patients with CLBP. The wearable device and a deep learning system will capture and analyze movement characteristics to identify completed exercises and protective behaviors, offering an objective measure of adherence while highlighting psychological factors such as fear and catastrophic thinking. [27] By detecting whether they complete the movement and whether there is a problem movement, the patient can be fed back to improve their exercise compliance. To support ease of use, the app has been streamlined, and dedicated training and phone support will be provided for older adults. Nicholl et al. [28] developed a related mobile app for LBP self-management as part of the self-BACK project, offering algorithm-based digital interventions. Our study, however, includes weekly video supervision and health coaching, and employs a wearable device capable of detecting specific movements and protective behaviors rather than only step counts, potentially offering more precise assessments of adherence and psychological shifts. Ultimately, this trial will compare a telemedicine-supported structured exercise program (TG, trial group) to traditional therapy (CG, control group) in reducing pain and activity limitation among patients with CLBP following conservative treatment discharge.

Material and methods

Aims of the study

Primary objective

The primary objective of this study is to assess the effect of a telemedicine-supported structured exercise program on physical function including pain in adult CLBP patients in

Information Classification: General

Chengdu, Sichuan Province, China, compared with traditional therapy (paper-based exercise therapy).

Secondary objective

1.To assess the impact of a telemedicine-supported structured exercise program on quality of life, and psychopathological symptoms in adult CLBP patients in Chengdu, Sichuan Province, China, compared with traditional therapy (paper-based exercise therapy).

2.To assess the impact of a telemedicine-supported structured exercise program on exercise compliance and pain fear or pain catastrophic imagery in adult CLBP patients in Chengdu, Sichuan Province, China, compared with traditional therapy (paper-based exercise therapy).

Study design

This is a randomized controlled trial with two parallel arms, conducted in an open-label, difference-test manner, following a 1:1 allocation ratio, within a single-center over a span of 8 weeks. The trial follows the recommendations of the Standard Protocol Items: Recommendations for Interventional Trials (SPIRIT) statement. [29] A completed SPIRIT checklist can be found in online supplemental material I. This study has received approval from the Ethics Committee of West China Hospital, Sichuan University. Any significant protocol modifications will be submitted to the Biomedical Ethics Committee of West China Hospital, Sichuan University for review. The study protocol has been registered at the Chinese Clinical Trial Registry (ChiCTR2300071560). The current study protocol is the sixth version updated on April 21, 2025.

Setting & Recruitment

Participants will be recruited from the Specialist Outpatient Clinic at West China Hospital, Sichuan University. Individuals who meet the eligibility criteria will be informed about the trial procedures and enrolled upon providing written informed consent; those who do not meet the criteria will receive standard treatment in accordance with the trial protocol. To ensure an

Information Classification: General

adequate sample size, standardized training and supervision will be provided to researchers, improving the success rate of informed consent acquisition. The trial aims to recruit 78 participants, and additional strategies (e.g., referrals from medical examination centers or relevant departments, poster campaigns) will be employed to facilitate timely enrollment. Recruitment will commence within one year of the scheduled enrollment date and will proceed based on site readiness. Details on data collection and intervention administration are available in Supplemental Material II. All eligible participants who agree to partake in the trial will be randomly assigned and briefed on the required procedures and written informed consent (accessible in Supplemental Material III) must be provided prior to enrollment. Baseline data for both groups will be gathered via a questionnaire administered before the intervention in Room 902 of the Special Needs Outpatient Department, collecting demographic information (e.g., gender, age, height, weight, occupation, education), potential causes of back pain, physical activity level, medical and exercise treatment history, duration of low back pain, severity of low back pain (current, worst, and least), level of functional impairment, and types of pain avoidance behaviors (see Supplemental Material II). Experienced physical therapists, who are independent of the trial design, intervention, and statistical analysis, will perform outcome measurements at baseline, after the 4-week intervention, and after the 8-week intervention, as illustrated in Figure 1.

Eligibility criteria and Consent

If a patient expresses interest in participating, researchers will evaluate their eligibility based on the study's inclusion and exclusion criteria (Table 1).[30] Both physicians and researchers will provide prospective participants with consent material detailing the context of the trial, the relevant population, interventions, risks and advantages of participating, along with rights and obligations. Individuals who agree to participate will be given thorough written information about the goals and methods of the study, and they will be asked to sign three informed consent forms. One copy will be kept by the participants, one will be electronically scanned on the first visit by the physician, and the third copy will be safely preserved by the Sichuan University Ethics Committee.

Information Classification: General

Randomization and blinding

Randomization will be conducted as participants are enrolled, using block randomization with a computer-generated random sequence (blocks of size four). A designated data manager, who is not involved in recruitment, intervention, or evaluation, will generate and securely store the random sequences in IBM SPSS, version 26.0.1. To conceal group allocation, an independent researcher not involved in the trial will place assignment codes into sequentially numbered, sealed, opaque envelopes according to the computer-generated random sequence. When each envelope is opened, participants will be assigned to either the control group or the trial group based on the corresponding “0” or “1” on the random number card. At enrollment, a physician not involved in randomization will assess potential participants, and the assignment group will be revealed onsite by opening the sealed envelopes, ensuring a 1:1 randomization ratio. Permuted blocks of size four will be used to maintain balanced allocation. Blinding physicians and volunteers to group assignments is not feasible, and participants are similarly unblinded due to self-reported outcomes. Assessors are also not blinded; however, to minimize bias, the trial hypothesis is not disclosed to either assessors or participants. The randomization list will remain secured (both physically and digitally), accessible only to the primary investigator and the designated data manager, thus maintaining its integrity throughout the study.

Interventions

The physiotherapists who delivered the intervention in both two groups will not be involved in the outcome measures and are not blind to the group assignment. The two groups will conduct an 8-week intervention, for a total of 24 sessions. The interventions in both groups will be completed over 8 weeks, following the 2020 clinical practice guidelines of the North American Spine Association.[31] Subjects will be encouraged to participate in the intervention and assessment throughout the trial protocol. Frequency of attendance, medication changes, adverse events, etc. will be reported by the participants to the physiotherapist in charge of the trial management and will be duly recorded in the case report form.

Information Classification: General

All participants will use the same version of the app throughout the trial. If a participant's condition worsens during treatment, they may seek other treatments (e.g., pharmacotherapy, physical therapy), but additional exercise therapy is prohibited. Participants must accurately report any extra treatments to the investigator, who will document them in the case report forms. To enhance adherence to intervention protocols, a weekly group WeChat video session will be held. This group-based health coaching allows participants to exchange peer support and psychological encouragement, thereby strengthening engagement between therapists and patients.

TG Therapy

For the trial group, the intervention consisted of app-based exercise therapy (40-minute sessions, three times per week for eight weeks), patient education (10-minute sessions, once weekly for eight weeks), and WeChat video-based health coaching (20-minute sessions, once weekly for eight weeks).

App-based exercise therapy

During the initial visit, the physician implements app-based exercise therapy in three parts: (1) guiding the patient through the "ShuKang PRO" app login, (2) formulating an individualized exercise prescription, and (3) demonstrating the exercises and providing instructions to the therapist (Figure 2). Table 2 outlines the specific exercises taught by the therapist responsible for the intervention. Exercise prescriptions are derived from baseline data and FITT-VP principles, primarily emphasizing core stability and motor control training recommended by clinical guidelines.[31] The physician adjusts the stepwise treatment plan according to individual variability.

To improve compliance with wearing the wearable device at home, a fabric fixture has been developed to hold the device nodes securely and ensure correct positioning. The wearable device uses noninvasive Inertial Measurement Units (IMUs) to capture body acceleration, angular velocity, and orientation without direct skin contact. Prior to public release, we will seek regulatory medical device approval to ensure convenience and comfort for daily use. By tracking motion

Information Classification: General

features, the device recognizes whether the prescribed movements have been completed. Before each session, patients can review the number of exercises, estimated duration, exercise preview, and written instructions. Upon reaching the upper or lower limit of the prescribed duration, the system alerts patients to begin a finishing stretch or end the exercise. Patients are then encouraged to aim for the targeted standard and are asked to fill in the Rating of Perceived Exertion (RPE) scale, which serves solely as feedback on exercise intensity and is not used as an outcome measure.[32] The physician modifies the prescription based on the principles of overload and gradual progression in exercise volume, by adjusting the duration or repetition of each exercise.[33]

Patients can schedule exercise times using the app's alarm function. Physicians may set phased goals in advance and send weekly reminders via the app to track goal completion. After being trained in all movements at the first consultation, participants enter an eight-week program comprising three weekly sessions of 40–60 minutes under a physical therapist's supervision. Each session consists of a warm-up, functional training, and relaxation. Table 2 details the primary exercises performed during the eight weeks, and the physician tailors intensity and difficulty according to each patient's characteristics.[34]

App-based patient education

During the baseline assessment, a physical therapist presents an animated educational video on chronic low back pain (CLBP). Once weekly, patients receive an illustrated educational piece about CLBP via the app, taking approximately five to ten minutes to read.[35] Topics include the definition, pathogenesis, etiology, and diagnosis of CLBP; management of daily activities; prevention of progression and recurrence; diet and weight control. Every Friday, patients complete a self-administered CLBP knowledge questionnaire in the app to verify comprehension, rather than for statistical analysis. Patients may retake the questionnaire until they select the correct responses.

WeChat video-based health coaching

A weekly group WeChat video session (40 minutes) is scheduled, such that eight of the 24 sessions are therapist supervised. During these sessions, the therapist reviews each patient's

Information Classification: General

condition, diet, and weight, and patients can share experiences and support each other. This group-based health coaching fosters peer support strengthens therapist-patient rapport and builds mutual trust. The weekly meeting also allows the therapist to confirm exercise accuracy, preventing new injuries. When a patient demonstrates correct movement execution and meets progress criteria, the therapist can adjust the treatment plan accordingly.

CG Therapy

The control group's interventions included paper-based exercise therapy and app-based patient education. At the initial diagnosis, physical therapists responsible solely for the intervention will provide patients with a printed exercise manual containing the same training exercises and frequency as the trial group, demonstrated once by the therapists. No further instruction will be offered during the subsequent eight weeks unless requested by the patient. The manual employs text and graphics to highlight essential elements, standards, and precautions for each movement. If patients encounter difficulties exercising at home, they may seek assistance at a designated partner community hospital or the main hospital, where physical therapists have received prior training. Over eight weeks, participants will complete 24 unsupervised self-training sessions (three times per week). In parallel, the control group will receive the same patient education content through an app that provides information once weekly for eight consecutive weeks. The app is configured only for patient education and does not grant access to the exercise training module.

Outcome measures

The outcome measures are divided into primary, secondary outcomes and other outcomes, which include pain intensity, physical function, quality of life, psychopathological symptoms, exercise compliance, fear-avoidance in exercise and catastrophizing, and adverse events. Table 3 provides an overview of the questionnaire items and the time points for outcome measurements. Changes in outcome measures will be primarily evaluated after 8 weeks of treatment.

Information Classification: General

Primary outcomes

CLBP severity will be measured using the Numerical Rating Scale (NRS), which ranges from 0 (no pain) to 10 (severe pain). Patients select a single number (0–10) that best represents their current level of pain. The NRS is widely used due to its simplicity, ease of administration, and reliable detection of pain severity.[36]

The Roland Morris Disability Questionnaire (RMDQ) evaluates functional status in patients with low back pain.[37] It consists of 24 items specifically related to limitations caused by low back pain. Each item is scored as 1 (yes) or 0 (no), yielding a total score between 0 and 24, where higher scores indicate greater functional impairment.

Secondary outcomes

The "Timed Up & Go" (TUG) test offers a rapid assessment of walking function.[38] Patients start seated with their backs against a chair, then stand up, walk three meters, turn around, return to the chair, and sit down. The time taken (in seconds) to complete this sequence is recorded. Lower times reflect better mobility, whereas higher times indicate mobility impairment and an increased risk of falling.[39]

The 12-item Short Form Health Survey (SF-12) measures eight dimensions of health-related quality of life.[40] These dimensions are summarized into two component scores: the Physical Component Summary (PCS) and the Mental Component Summary (MCS). The SF-12 is shorter and easier to administer than the original SF-36, while retaining good reliability and validity.

The Depression-Anxiety-Stress Scale (DASS21) evaluates mental health based on depression, anxiety, and stress factors.[41] It employs a 4-point Likert scale (0–3) to indicate how frequently participants experience negative emotional states. Higher scores signify more severe psychopathological symptoms.

The Exercise Adherence Rating Scale (EARS) assesses adherence to exercise programs prescribed for individuals with chronic pain.[42] It contains 16 items grouped into three subscales that capture self-reported adherence and the reasons for compliance or non-

Information Classification: General

compliance. Wearable devices will also be used to calculate completion rates of exercise prescriptions among patients with CLBP.

Other outcomes

Wearable devices will detect the frequency of protective behaviors associated with fear-avoidance and catastrophizing. A decrease in these behaviors indicates reduced fear of movement and pain-related catastrophizing.[27,43,44]

An adverse event is defined as any issue involving the lower back or other parts of the body that the participant perceives to be related to the trial, meeting one of the following criteria: i) that caused the participant to seek medical care for at least two days; and ii) that made their pain or impairment worse.[45] After the intervention, participants will be surveyed regarding any adverse events.

Criteria for discontinuing or modifying allocated interventions

If a participant opts to withdraw from the trial prematurely, the physiotherapist will promptly contact them to determine the reason for withdrawal. Participants who exit the trial, become unreachable for over two weeks, and fail to complete the outcome evaluation will be categorized as dropouts.[46]

1) Patients desiring to cease participation.

2) Patients unable to undergo the baseline assessment.

3) Patients failing to complete the app-based exercise therapy sessions.

4) Patients experiencing exacerbated symptoms.

Sample size

The sample size was calculated using G*Power 3.1.9 under the following conditions: based on Cohen's standard for effect sizes, 0.2, 0.5, and 0.8 indicate small, medium, and large effects, respectively.[47] A value of 0.3 was selected to represent a small-to-medium effect size, drawing on Murtezani's findings.[48] With $\alpha = 0.05$, power = 0.8, and a correlation among repeated

Information Classification: General

measures of 0.5, a two-way repeated measures analysis of variance was planned, featuring three measurement points for each of the two groups. The resulting sample size was 58, and accounting for a 25% dropout rate increased the required sample size to 78 (total).

Statistical analysis

All analyses will follow the Consolidated Standards of Reporting Trials (CONSORT) guidelines.[49] Baseline measurements, group comparability, and descriptive characteristics will be assessed. Because patient dropout can occur for various reasons, data analyses will include both intention-to-treat (ITT) and per-protocol (PP) approaches. PP analysis considers only participants who complete the entire trial post-randomization, whereas ITT analysis includes follow-up data from all randomized participants.[50] While PP analysis can exaggerate between-group differences, ITT analysis minimizes bias and loss to follow-up, generally producing more conservative estimates. Therefore, ITT analysis will be used as the primary method to prevent overstating effectiveness. The primary endpoint of interest is assessed after eight weeks of treatment.

All statistical analyses will be performed using IBM SPSS, version 26.0.1, with two-tailed tests (α=0.05) determining statistical significance. At baseline, categorical variables (e.g., sex, occupation, education level) will be compared between trial and control groups using chi-square tests. Continuous variables (e.g., age, BMI, pain intensity, duration of low back pain) will undergo a normality test. If the data follow a normal distribution, results will be expressed as mean ± standard deviation (SD) and analyzed using independent-samples T-tests or Mann-Whitney U tests. Otherwise, the median (interquartile range) will be reported, and nonparametric tests will be applied. If the data meet the assumptions of homogeneity of variance and sphericity, a two-factor, three-level repeated-measures analysis of variance (group×time) will be conducted; otherwise, a mixed-effects model will be used.

According to the International Spine Conference, a two-point change in pain intensity (NRS scale: 0–10) signifies a minimum clinically important difference (MCID).[51] Chi-square tests will compare the proportion of patients in each group who achieve MCID. Cohen's criteria will be used to evaluate the effect size across all outcome measures.[47] No interim or subgroup analyses

Information Classification: General

are planned. An ITT approach will be used to address missing data for the primary outcome, and multiple imputation will minimize missing values.

Data management and auditing

Data will be collected both on paper and electronically. If participants return for follow-up, they will complete paper questionnaires; otherwise, they will use online forms. Paper surveys will be administered by an assessor who has received standardized training, and patients will also fill out their own paper questionnaires. Online surveys will be delivered through a designated app, and TUG testing will be conducted via video conferencing. Both groups may be provided with wearable devices for home use, which will be collected at the end of the intervention. A statistician not involved in the trial intervention will retrieve wearable device data, which two data administrators will then enter into anonymized databases equipped with logic checks (e.g., mandatory fields, response limits). These data will be organized into tables for statistical analysis. During recruitment, patients will receive comprehensive information about the trial's requirements and design, and they may withdraw at any time without giving a reason. Their progress will be tracked using a quality form, and those who drop out or discontinue the intervention will also have outcome data collected. Outcome assessors will document experimental procedures and data on a case report form, assigning each participant a unique code to protect privacy; only the assessors and the corresponding author will have access to these forms. All data entries will be verified twice by two independent assessors, after which the entries become non-editable. A Data & Safety Monitoring Committee is not required, given the low-risk interventions and the minimal likelihood of serious adverse events (incapacitating, life-threatening, hospitalization, or death). Auditing will involve on-site visits by independent supervisors twice a month to verify the presence and accuracy of investigation files, as well as checks on 20% of participants chosen at random to review source data, inclusion and exclusion criteria, missing scales, reporting, and informed consent.

Discussion

The primary objective of this study is to compare the efficacy of a telemedicine-supported structured exercise program with a paper-based exercise therapy program in improving pain among patients with CLBP in Chengdu, Sichuan Province, China. Current international guidelines strongly recommend exercise and behavioral therapy as first-line treatments for persistent chronic pain, emphasizing the importance of patient self-management.[52] In line with these recommendations, the telemedicine-supported TG focuses on establishing a home-based self-management model.

Telemedicine replaces face-to-face physical therapist guidance with app-based monitoring, wearable devices, and the WeChat platform, thereby reducing time and geographic constraints while allowing patients to perform sustainable home-based exercises.[53] This approach is reinforced by the pioneering use of wearable devices, which objectively document exercise duration (reflecting adherence) and capture patients' behavioral patterns. By drawing on diagnostic opinions from multiple pain experts, the wearable system can recommend personalized exercise regimens for CLBP patients. Changes in behavioral patterns before and after treatment serve as an objective indicator of therapeutic efficacy. The deep learning system can also track shifts in pain perception and fear of movement by monitoring protective behaviors, while weekly WeChat conferences bolster patients' trust in therapists and encourage adherence through group interactions. Patient education aims to alter pain-related beliefs, highlight the importance of healthy habits for pain relief, and promote an active lifestyle to manage ongoing CLBP progression.[54] Beyond pain and physical function, this study also assesses pain cognition to capture a comprehensive view of outcomes.

In conclusion, this trial will investigate the impact of a telemedicine-supported structured exercise program on patients with CLBP in Chengdu, Sichuan Province, China. The results may offer a more effective home-based self-management model, ease the medical burden of CLBP, and inform public health and preventative strategies worldwide.[55] The long-term sustainability and cost-effectiveness of telemedicine supported interventions will be the focus of future research.

Information Classification: General

Acknowledgments

The authors are grateful to Hui-Zhen Liu and colleagues from Center of Biostatistics、Design、Measurement and Evaluation (CBDME) of West China Hospital for providing critical comments on the study design, sample size calculations, and for help in developing a statistical analysis plan. We would like to thank the technical support of the ShuKang company and Chong-Yang Wang team of Tsinghua University.

Author contributions

All authors made a significant contribution to the work reported, whether that is in the conception, study design, execution, acquisition of data, analysis and interpretation, or in all these areas; took part in drafting, revising or critically reviewing the article; gave final approval of the version to be published; have agreed on the journal to which the article has been submitted; and agree to be accountable for all aspects of the work.

Competing interests

None declared.

Data sharing

There are no plans for granting public access to the full protocol, participant-level dataset, and statistical code.

Funding statement

This study was supported by the National Natural Science Foundation of China (82272599), Natural Science Foundation of Sichuan Province (2024NSFSC0533), Sichuan University “Research Special Project on the Comprehensive Reform of Innovative Educational Practices Enabled by Artificial Intelligence” (2024-44), 1.3.5 project for disciplines of excellence,

Information Classification: General

West China Hospital, Sichuan University (ZYGD23014), “Qimingxing” Research Fund for Young Talents of West China Hospital (HXQMX0113), Sichuan University West China School of Medicine Graduate Education Reform Project (HXYJS202415) and Science and Technology Bureau of Deyang City (Deyang Clinical Research Center for Rehabilitation Medicine, Mianzhu People's Hospital, 2023-64). The funders played no role in the design, conduct, or reporting of this study.

Patient and public involvement

During the study design, patients were consulted to assess their confidence and expectations regarding telemedicine-based home rehabilitation. Feedback from a small group of patients informed the selection of primary outcomes and other study components. An advisory group was formed at the outset of the project, and investigators at each site are responsible for participant recruitment and trial execution.

Provisions for post-trial care

If risks arise during treatment, the project team will address study-related adverse events at the participant’s expense. Should a serious adverse event be deemed related to the clinical research, the project team will cover the necessary treatment costs. Participants who can provide legally valid evidence of loss will receive appropriate financial compensation, including a 300-yuan subsidy for travel and housing expenses.

Confidentiality

All research data will be labeled with a unique participant identification code. Only the study team will have access to the key linking these codes to individual identities. After the study concludes, the principal investigator will store and secure the code key in accordance with research guidelines. No patient-identifiable information will appear in any publications.

Information Classification: General

Consent for publication

Written informed consent will be obtained from the patient for publication of this RCT and any accompanying images. A copy of the written consent is available for review by the Editor-in-Chief of this journal.

References

1. Dionne CE, Dunn KM, Croft PR, et al. A consensus approach toward the standardization of back pain definitions for use in prevalence studies. *Spine (Phila Pa 1976)*. Jan 1 2008;33(1):95-103. doi:10.1097/BRS.0b013e31815e7f94
2. Williams CM, Maher CG, Latimer J, et al. Efficacy of paracetamol for acute low-back pain: a double-blind, randomised controlled trial. *Lancet*. Nov 1 2014;384(9954):1586-96. doi:10.1016/s0140-6736(14)60805-9
3. Global burden of 369 diseases and injuries in 204 countries and territories, 1990-2019: a systematic analysis for the Global Burden of Disease Study 2019. *Lancet*. Oct 17 2020;396(10258):1204-1222. doi:10.1016/s0140-6736(20)30925-9
4. Hoy D, March L, Brooks P, et al. The global burden of low back pain: estimates from the Global Burden of Disease 2010 study. *Ann Rheum Dis*. Jun 2014;73(6):968-74. doi:10.1136/annrheumdis-2013-204428
5. Knezevic NN, Candido KD, Vlaeyen JWS, Van Zundert J, Cohen SP. Low back pain. *Lancet*. Jul 3 2021;398(10294):78-92. doi:10.1016/s0140-6736(21)00733-9
6. Jack K, McLean SM, Moffett JK, Gardiner E. Barriers to treatment adherence in physiotherapy outpatient clinics: a systematic review. *Man Ther*. Jun 2010;15(3):220-8. doi:10.1016/j.math.2009.12.004
7. Bodes Pardo G, Lluch Girbés E, Roussel NA, Gallego Izquierdo T, Jiménez Penick V, Pecos Martín D. Pain Neurophysiology Education and Therapeutic Exercise for Patients With Chronic Low Back Pain: A Single-Blind Randomized Controlled Trial. *Arch Phys Med Rehabil*. Feb 2018;99(2):338-347. doi:10.1016/j.apmr.2017.10.016
8. Puentedura EJ, Flynn T. Combining manual therapy with pain neuroscience education in the treatment of chronic low back pain: A narrative review of the literature. *Physiother Theory Pract*. Jul 2016;32(5):408-14. doi:10.1080/09593985.2016.1194663
9. Tedeschi R, Giorgi F, Platano D, Berti L. Classifying Low Back Pain Through Pain Mechanisms: A Scoping Review for Physiotherapy Practice. *J Clin Med*. Jan 10 2025;14(2)doi:10.3390/jcm14020412
10. Clael S, Campos LF, Correia KL, et al. Exercise interventions can improve muscle strength, endurance, and electrical activity of lumbar extensors in individuals with non-specific low back pain: a systematic review with meta-analysis. *Sci Rep*. Aug 19 2021;11(1):16842. doi:10.1038/s41598-021-96403-7
11. Hayden JA, Ellis J, Ogilvie R, et al. Some types of exercise are more effective than others in people with chronic low back pain: a network meta-analysis. *J Physiother*. Oct 2021;67(4):252-262. doi:10.1016/j.jphys.2021.09.004
12. Owen PJ, Miller CT, Mundell NL, et al. Which specific modes of exercise training are most effective for treating low back pain? Network meta-analysis. *Br J Sports Med*. Nov 2020;54(21):1279-1287. doi:10.1136/bjsports-2019-100886
13. Meints SM, Edwards RR. Evaluating psychosocial contributions to chronic pain outcomes. *Prog Neuropsychopharmacol Biol Psychiatry*. Dec 20 2018;87(Pt B):168-182. doi:10.1016/j.pnpbp.2018.01.017
14. Pinto RZ, Ferreira PH, Kongsted A, Ferreira ML, Maher CG, Kent P. Self-reported moderate-to-vigorous leisure time physical activity predicts less pain and disability over 12

Information Classification: General

months in chronic and persistent low back pain. *Eur J Pain*. Sep 2014;18(8):1190-8. doi:10.1002/j.1532-2149.2014.00468.x

15. Lin CC, McAuley JH, Macedo L, Barnett DC, Smeets RJ, Verbunt JA. Relationship between physical activity and disability in low back pain: a systematic review and meta-analysis. *Pain*. Mar 2011;152(3):607-613. doi:10.1016/j.pain.2010.11.034

16. Kemler E, Noteboom L, van Beijsterveldt AM. Characteristics of Fitness-Related Injuries in The Netherlands: A Descriptive Epidemiological Study. *Sports (Basel)*. Nov 22 2022;10(12)doi:10.3390/sports10120187

17. Matarán-Peñarrocha GA, Lara Palomo IC, Antequera Soler E, et al. Comparison of efficacy of a supervised versus non-supervised physical therapy exercise program on the pain, functionality and quality of life of patients with non-specific chronic low-back pain: a randomized controlled trial. *Clin Rehabil*. Jul 2020;34(7):948-959. doi:10.1177/0269215520927076

18. Palazzo C, Klinger E, Dorner V, et al. Barriers to home-based exercise program adherence with chronic low back pain: Patient expectations regarding new technologies. *Ann Phys Rehabil Med*. Apr 2016;59(2):107-13. doi:10.1016/j.rehab.2016.01.009

19. Wolever RQ, Eisenberg DM. What is health coaching anyway?: standards needed to enable rigorous research. *Arch Intern Med*. Dec 12 2011;171(22):2017-8. doi:10.1001/archinternmed.2011.508

20. Duong V, Robbins SR, Dennis S, Venkatesha V, Ferreira ML, Hunter DJ. Combined Digital Interventions for Pain Reduction in Patients Undergoing Knee Replacement: A Randomized Clinical Trial. *JAMA Netw Open*. Sep 5 2023;6(9):e2333172. doi:10.1001/jamanetworkopen.2023.33172

21. Gunter RL, Chouinard S, Fernandes-Taylor S, et al. Current Use of Telemedicine for Post-Discharge Surgical Care: A Systematic Review. *J Am Coll Surg*. May 2016;222(5):915-27. doi:10.1016/j.jamcollsurg.2016.01.062

22. Svendsen MJ, Wood KW, Kyle J, et al. Barriers and facilitators to patient uptake and utilisation of digital interventions for the self-management of low back pain: a systematic review of qualitative studies. *BMJ Open*. Dec 12 2020;10(12):e038800. doi:10.1136/bmjopen-2020-038800

23. Hewitt S, Sephton R, Yeowell G. The Effectiveness of Digital Health Interventions in the Management of Musculoskeletal Conditions: Systematic Literature Review. *J Med Internet Res*. Jun 5 2020;22(6):e15617. doi:10.2196/15617

24. Bailey JF, Agarwal V, Zheng P, et al. Digital Care for Chronic Musculoskeletal Pain: 10,000 Participant Longitudinal Cohort Study. *J Med Internet Res*. May 11 2020;22(5):e18250. doi:10.2196/18250

25. Tedeschi R, Platano D, Pillastrini P, Berti L, Benedetti MG. Effectiveness of tele-rehabilitation in patients with knee osteoarthritis: A randomized controlled trial. *Digit Health*. Jan-Dec 2024;10:20552076241286186. doi:10.1177/20552076241286186

26. Lewkowicz D, Slosarek T, Wernicke S, Winne A, Wohlbrandt AM, Bottinger E. Digital Therapeutic Care and Decision Support Interventions for People With Low Back Pain: Systematic Review. *JMIR Rehabil Assist Technol*. Nov 19 2021;8(4):e26612. doi:10.2196/26612

27. Wang C, Gao Y, Mathur A, Williams ACDC, Lane ND, Bianchi-Berthouze N. Leveraging Activity Recognition to Enable Protective Behavior Detection in Continuous Data. *Proc ACM Interact Mob Wearable Ubiquitous Technol*. 2021;5(2):Article 81. doi:10.1145/3463508

28. Sandal LF, Stochkendahl MJ, Svendsen MJ, et al. An App-Delivered Self-Management Program for People With Low Back Pain: Protocol for the selfBACK Randomized Controlled Trial. *JMIR Res Protoc*. Dec 3 2019;8(12):e14720. doi:10.2196/14720

29. Chan AW, Tetzlaff JM, Altman DG, et al. SPIRIT 2013 statement: defining standard protocol items for clinical trials. *Ann Intern Med*. Feb 5 2013;158(3):200-7. doi:10.7326/0003-4819-158-3-201302050-00583

30. Masood R, Mandalia K, Moverman MA, et al. Patients With Functional Somatic Syndromes-Fibromyalgia, Irritable Bowel Syndrome, Chronic Headaches, and Chronic Low Back Pain-Have Lower Outcomes and Higher Opioid Usage and Cost After Shoulder and Elbow Surgery. *Arthroscopy*. Jun 2023;39(6):1529-1538. doi:10.1016/j.arthro.2022.12.028

31. Kreiner DS, Matz P, Bono CM, et al. Guideline summary review: an evidence-based clinical guideline for the diagnosis and treatment of low back pain. *Spine J*. Jul 2020;20(7):998-1024. doi:10.1016/j.spinee.2020.04.006

Information Classification: General

32. Dishman RK. Prescribing exercise intensity for healthy adults using perceived exertion. *Med Sci Sports Exerc*. Sep 1994;26(9):1087-94.
33. Ibrahim AA, Akindele MO, Ganiyu SO. Motor control exercise and patient education program for low resource rural community dwelling adults with chronic low back pain: a pilot randomized clinical trial. *J Exerc Rehabil*. Oct 2018;14(5):851-863. doi:10.12965/jer.1836348.174
34. !!! INVALID CITATION !!! [32-34];
35. Halliday MH, Pappas E, Hancock MJ, et al. A randomized clinical trial comparing the McKenzie method and motor control exercises in people with chronic low back pain and a directional preference: 1-year follow-up. *Physiotherapy*. Dec 2019;105(4):442-445. doi:10.1016/j.physio.2018.12.004
36. !!! INVALID CITATION !!! [36];
37. Yi H, Ji X, Wei X, et al. Reliability and validity of simplified Chinese version of Roland-Morris questionnaire in evaluating rural and urban patients with low back pain. *PLoS One*. 2012;7(1):e30807. doi:10.1371/journal.pone.0030807
38. Elsner VR, Trevizol L, de Leon I, et al. Therapeutic effectiveness of a single exercise session combined with WalkAide functional electrical stimulation in post-stroke patients: a crossover design study. *Neural Regen Res*. May 2021;16(5):805-812. doi:10.4103/1673-5374.297078
39. van Lummel RC, Walgaard S, Hobert MA, et al. Intra-Rater, Inter-Rater and Test-Retest Reliability of an Instrumented Timed Up and Go (iTUG) Test in Patients with Parkinson's Disease. *PLoS One*. 2016;11(3):e0151881. doi:10.1371/journal.pone.0151881
40. Ware J, Jr., Kosinski M, Keller SD. A 12-Item Short-Form Health Survey: construction of scales and preliminary tests of reliability and validity. *Med Care*. Mar 1996;34(3):220-33. doi:10.1097/00005650-199603000-00003
41. Lovibond PF, Lovibond SH. The structure of negative emotional states: comparison of the Depression Anxiety Stress Scales (DASS) with the Beck Depression and Anxiety Inventories. *Behav Res Ther*. Mar 1995;33(3):335-43. doi:10.1016/0005-7967(94)00075-u
42. Newman-Beinart NA, Norton S, Dowling D, et al. The development and initial psychometric evaluation of a measure assessing adherence to prescribed exercise: the Exercise Adherence Rating Scale (EARS). *Physiotherapy*. Jun 2017;103(2):180-185. doi:10.1016/j.physio.2016.11.001
43. Mesaroli G, Vader K, Rosenbloom BN, Birnie KA, Stinson J. Sensibility and measurement properties of the Tampa Scale of Kinesiophobia to measure fear of movement in children and adults in surgical settings. *Disabil Rehabil*. Jul 2023;45(14):2390-2397. doi:10.1080/09638288.2022.2090624
44. Shen B, Wu B, Abdullah TB, et al. Translation and validation of Simplified Chinese version of the Pain Catastrophizing Scale in chronic pain patients: Education may matter. *Mol Pain*. Jan-Dec 2018;14:1744806918755283. doi:10.1177/1744806918755283
45. Hinman RS, Kimp AJ, Campbell PK, et al. Technology versus tradition: a non-inferiority trial comparing video to face-to-face consultations with a physiotherapist for people with knee osteoarthritis. Protocol for the PEAK randomised controlled trial. *BMC Musculoskelet Disord*. Aug 7 2020;21(1):522. doi:10.1186/s12891-020-03523-8
46. Li F, Harmer P, Fitzgerald K, et al. Tai chi and postural stability in patients with Parkinson's disease. *N Engl J Med*. Feb 9 2012;366(6):511-9. doi:10.1056/NEJMoa1107911
47. Faul F, Erdfelder E, Lang AG, Buchner A. G*Power 3: a flexible statistical power analysis program for the social, behavioral, and biomedical sciences. *Behav Res Methods*. May 2007;39(2):175-91. doi:10.3758/bf03193146
48. Murtezani A, Govori V, Meka VS, Ibraimi Z, Rrecaj S, Gashi S. A comparison of mckenzie therapy with electrophysical agents for the treatment of work related low back pain: A randomized controlled trial. *J Back Musculoskelet Rehabil*. 2015;28(2):247-53. doi:10.3233/bmr-140511
49. !!! INVALID CITATION !!! [49];
50. Zhang H, Xin H, Du Y, et al. Tuberculosis preventive treatment among individuals with inactive tuberculosis suggested by untreated radiographic abnormalities: a community-based randomized controlled trial. *Emerg Microbes Infect*. Dec 2023;12(1):e2169195. doi:10.1080/22221751.2023.2169195

Information Classification: General

51. Ostelo RW, Deyo RA, Stratford P, et al. Interpreting change scores for pain and functional status in low back pain: towards international consensus regarding minimal important change. *Spine (Phila Pa 1976)*. Jan 1 2008;33(1):90-4. doi:10.1097/BRS.0b013e31815e3a10
52. Cohen KR. Management of Chronic Low Back Pain. *JAMA Intern Med*. Feb 1 2022;182(2):222-223. doi:10.1001/jamainternmed.2021.7359
53. Marcuzzi A, Nordstoga AL, Bach K, et al. Effect of an Artificial Intelligence-Based Self-Management App on Musculoskeletal Health in Patients With Neck and/or Low Back Pain Referred to Specialist Care: A Randomized Clinical Trial. *JAMA Netw Open*. Jun 1 2023;6(6):e2320400. doi:10.1001/jamanetworkopen.2023.20400
54. Bernstein IA, Malik Q, Carville S, Ward S. Low back pain and sciatica: summary of NICE guidance. *Bmj*. Jan 6 2017;356:i6748. doi:10.1136/bmj.i6748
55. Foster NE, Anema JR, Cherkin D, et al. Prevention and treatment of low back pain: evidence, challenges, and promising directions. *Lancet*. Jun 9 2018;391(10137):2368-2383. doi:10.1016/s0140-6736(18)30489-6

Information Classification: General

Table 1 Inclusion and exclusion criteria

Inclusion criteria	Exclusion criteria
All applicants must be between 18 and 65 years old.	Suffering from a specific disease of the spine, such as infection, spinal tumor, spinal tuberculosis, fracture, spondylolisthesis, isthmus, or aneurysm.
Pain, and muscle stiffness are located below the coastal margin and above the gluteal fissure fold, with or without lower limb pain.	Pain caused by other diseases.
Pain lasts 12 weeks or more.	Cognitive impairment results in the inability to understand the physical therapist's instructions and the content of the app.
Pain intensity (when the worst pain) ≥ 3 on the Numeric Rating Scale (NRS).	Pregnant or breastfeeding.
Ability to operate a smartphone.	History of spine surgery.
Voluntarily participate in the trial and sign the informed consent.	Patients who have received exercise therapy in the past three months for low back pain.
Accepted randomization.	Patients with severe cardiovascular and cerebrovascular diseases.
Candidates who meet all the above criteria will be included	Candidates meeting any of the above criteria will be excluded

Information Classification: General

Table 2 Core stability training & Motor control training

Exercise（R=Repetition, S=Second）	Sets and Repetitions per Week			
	1-4 Weeks		5-8 Weeks	
	Set	R/S	Set	R/S
Core stability training				
Abdominal breathing in the supine position (R)	3	5	3	10
Dynamic hip bridge (R)	3	10	3	15
Side bridge (S)	5	10	5	20
Plank support (S)	5	10	5	20
Prone position contralateral extension (R)	3	10	3	15
Four-point kneeling position alternate shoulder forward flexion (R)	3	10	3	15
Four-point kneeling contralateral extension (R)	3	10	3	15
Dead insect extension on the opposite side in a supine position (R)	3	5	3	10
Motor control training				
Pelvis forward and backward tilt in the supine position (S)	5	10	5	20
Stand on one leg with eyes closed (S)	3	5	3	10
Side leg lifting with one leg (R)	3	10	3	15
Single leg micro squat (R)	3	5	3	10
Squat (R)	3	5	3	10

Table 3 Overview of the measurements and timing of measurements

Domain	Reporting Method	Questionnaire	Baseline	4 weeks	8 weeks
Demographic	Self-reported	Purpose built	√	-	-

Information Classification: General

characteristics					
Pain intensity	Self-reported	NRS	√	√	√
Physical function	Self-reported	RMDQ	√	√	√
	Walking ability	TUG	√	√	√
Quality of life	Self-reported	SF-12	√	√	√
Psychopathological symptoms	Self-reported	DASS21	√	√	√
	Self-reported	EARS	-	√	√
Exercise compliance	Events	Movement completion rate	√	√	√
Fear-avoidance in exercise & catastrophization	Events	Frequency of protective behaviors	√	√	√
Adverse Events	Self-reported	-	-	√	√

Information Classification: General

Notes:

Abbreviations: LBP, Low Back Pain; CLBP, Chronic Low Back Pain; RCTs, Randomized Controlled Trials; TG, Trial Group; CG, Control Group; FITT-VP, Frequency, Intensity, Time, Type, Volume, Progression; IMUs, Inertial Measurement Units; RPE, Rating of perceived exertion; NRS, Numerical Rating Scale; RMDQ, Roland Morris Disability Questionnaire; TUG, Time up and go; SF-12, 12–item Short Form Health Survey; DASS21, Depression-Anxiety-Stress Scale; EARS, Exercise Adherence Rating Scale; SPIRIT, Standard Protocol Items: Recommendations for Interventional Trials; BMI, Body Mass Index; ITT, intention-to-treat; PP, Per-Protocol; SD, Standard Deviation; MCID, Minimum Clinically Important Difference

Information Classification: General

Figure Legends

Figure 1. Flow chart of the study

Figure 2. Intervention process at first visit.

Information Classification: General
