# Supplementary material for: Effect of telemedicine-supported structured exercise program in patients with chronic low back pain: a randomized controlled trial
Source: PLoS One. 2025 Jun 25;20(6):e0326218. doi: 10.1371/journal.pone.0326218 (PMC12193851; doi:10.1371/journal.pone.0326218)
Supplement: S2 File — (PDF) [file pone.0326218.s002.pdf]

Patient Consent Form

Dear participant:

You are invited to participate in the project "clinical research on digital exercise therapy for chronic low back pain" approved by the project of Discipline Excellence Development 1· 35 ·5. The study will be carried out at Sichuan University West China Hospital, and it is estimated that 78 participants will voluntarily participate. This study has been reviewed and approved by the Biomedical Ethics Review Committee of West China Hospital, Sichuan University (Review (1976) for 2022).

1. Why do we carry out this study?

Low back pain (LBP) is a leading cause of disability worldwide, and it is pain located between the lower costal margin and the upper gluteal fold, with or without pain in the legs. Chronic low back pain is defined when it persists for more than three months. Eighty percent of people will experience LBP at least once in their lifetime, about 50% of patients will fully recover within 2-3 weeks, and the rest will gradually develop CLBP. CLBP is the leading cause of disability years of life in both developed and developing countries and ranks sixth in the overall burden of disease. The reported annual prevalence rate of adult LBP is 20.88% ~ 29.88%, which has become a serious public health problem in China. Therefore, as a major global public health problem, CLBP has brought serious impacts on people's life and work, including pain, limited function, anxiety and depression, and reduced working hours.

The newly released expert consensus of China's Pain Department on chronic low back pain rehabilitation points out that exercise therapy can promote the health of lumbago muscles, reshape the function of lumbago, and reduce pain. Highly recommended exercise regimens include trunk strength training, hip strength training, core stability training/motor control, McKenzie mechanical diagnostic therapy

techniques, water exercises, yoga, Pilates, cardio, and walking exercises. International systematic reviews of high quality also show that trunk strength training, McKenzie mechanics techniques, core stability training, motor control and exercise therapy such as yoga and Pilates reduce pain better than non-exercise training controls. Owen et al included 89 randomized controlled trials and found low quality evidence that Pilates, core stability training, motor control training, resistance training, and aerobic exercise were the most effective treatment modalities. However, a structured exercise program cannot be done without the guidance of a professional rehabilitator. In China, most CLBP patients adopt outpatient treatment, but the treatment time is quite limited. A few patients cannot receive outpatient treatment due to remote locations, inconvenient transportation, and economic factors, so CLBP patients often need to exercise at home.

Since the outbreak of COVID-19, patients with chronic low back pain have also been limited in seeking outpatient treatment. Therefore, self-management of pain at home for CLBP patients has become a trend. Some studies have explored that unsupervised self-management at home is effective in improving the pain and disability of CLBP patients. Compared with the supervised physical therapy program, patients in the unsupervised group were less effective and compliant with at-home exercise, but the difference in efficacy between the two groups was not statistically significant. A study of semi-structured interviews found that relatively young patients prefer visual and dynamic support, and relatively old patients prefer guidance and supervision when exercising. Considering that without professional guidance and supervision, self-home exercise may cause harm and lead to reduced efficacy, it is urgent to find a better health service model that can meet patients' needs.

Digital exercise therapy is a new telemedicine technology based on the Internet, Internet of Things, mobile devices, smart phones, websites and other devices in the process of prevention, treatment, promotion and maintenance of health. Digital therapies can reduce health care costs, personalize content to meet patients' specific needs, and enable healthcare providers to overcome spatial and temporal barriers by

using information technology to track patients' disease changes, provide real-time guidance to patients, and collaborate with patients.

Therefore, an 8-week randomised controlled trial was designed to monitor the home exercise of CLBP patients based on an APP and a heart rate band, compared with an unsupervised paper version of self-exercise at home. To explore the impact of digital health-based exercise therapy on the physical function of patients with chronic low back pain, focusing on the improvement of patients' pain, mental health, quality of life and walking ability, aiming at the background of COVID-19 and the era of big data, to provide a more convenient, low consumption and effective method for the clinical treatment of patients with CLBP, promote the self-home management of patients with CLBP.

2. What do you need to do if you agree to participate in this study?

The study will last for 8 weeks, and the patients will be randomly assigned to two groups. You will be informed which group you belong to. The interventions in both groups were evidence-based therapies that did not involve invasive procedures.

1) After you sign the informed consent, you need to truthfully answer the questions about your basic information, including your age, height, weight, etc., as well as the general information about CLBP. We promise that your personal information and health condition will be kept strictly confidential and will only be used as the basis for judging your basic situation and as the basis for scientific research reports.
2) After the completion of basic data collection and baseline measurement, you will be randomly assigned to two groups to receive 8-week digital exercise therapy and paper exercise therapy intervention respectively.
3) You must be supervised and taken care of by other family members during exercise training, so as to ensure your safety during exercise training. If you are an elderly person living alone, we will arrange a nearby community medical institution to provide care for you during exercise according to the circumstances.
4) Exercise training three times a week, each time for 40-60 minutes, follow the exercise according to the APP/ paper sports video and graphic guidance.

Conduct self-home exercise under the supervision of family members. For the first intervention, you need to have an offline evaluation, discussion, and decision on a personalized exercise training program with physical therapists, and receive personalized exercise guidance. For the first intervention, having your family members supervise or take care of you is better.

5) During the 8-week intervention period, you need to receive the graphic patient education pushed by the APP once a week. We will provide you with educational information related to CLBP by using professional check forms and according to authoritative clinical guidelines.
6) During the 8-week intervention period, you need to receive WeChat video health guidance interaction once a week to help evaluate your weekly exercise progress and guide your lifestyle. The instructors will receive strict and professional training on chronic low back pain.
7) The exercise program you use, your feelings during exercise training, and other research-related contents should be kept in strict confidence from other patients and clinical evaluators participating in the study, and you should also inform your family members to keep in strict confidence.
8) In weeks 0, 4 and 8, you must be evaluated by the clinical evaluator through the APP, and truthfully fill in the questionnaire or answer the evaluator's questions. Each evaluation will take about 20-30 minutes. Your feelings and evaluation results during the evaluation should be kept confidential from the physical therapist and other participants.
9) During the 8-week intervention period, other treatments outside the study intervention (such as non-study drug therapy, physical therapy, etc.) will not be affected. You need to report to the clinical coordinator or physical therapist the time, method, and dosage of the therapy used.
10) During the entire study period, you will participate voluntarily. If you withdraw during the study due to personal reasons, you will need to report and explain to the clinical coordinator.

11) Throughout the study period, if you have any conditions lasting more than two days that require additional treatment, you should report them to your physiotherapist, clinical Coordinator (tel: 18810218262) or program contact person (tel: 18991463887).
12) For any health-related emergencies during the study period, you will need to report the events, timing, and treatment/management to the Clinical Coordinator (tel: 18810218262) after reasonable assistance or management.

3. What are the treatment options available?

During the intervention period, no treatment other than intervention is provided in this study, and subjects may choose to use other treatments other than exercise therapy, but subjects should report their use of other treatments to the clinical research assistant.

4. These individuals are not suitable for participating in this trial:

If you meet any of the following criteria, you are not eligible to participate in this study:

1) Having a specific disease of the spine, such as infection, spinal tumor, spinal tuberculosis, fracture, spondylolisthesis, isthmus, or aneurysm.
2) Pain caused by other diseases.
3) Mental illness or cognitive impairment (MMSE<24).
4) Pregnancy or breastfeeding.
5) History of spinal surgery.
6) Patients who have received exercise therapy for low back pain within the last three months.
7) Patients with severe cardiovascular and cerebrovascular diseases.

5. What are the potential risks and possible adverse events of participating in the study?

5.1 Risks associated with the intervention involved in this clinical trial mainly include:

5.1.1 According to the whole process involved in exercise intervention, directly relevant risks include:

1) Inadequate warm-up before exercise leads to muscle strain and other sports injuries.

2) Dizziness, chest pain, sudden cardiovascular and cerebrovascular diseases caused by unreasonable progression or weight bearing during exercise, or falls, fractures and other conditions caused by irregular exercise methods or movements.
3) Symptoms may increase and muscle soreness may occur after exercise;

5.1.2 According to the location and method of exercise intervention, the relevant risks include:

1) The main risks of exercise training under the supervision of physical therapists in the hospital are the three aspects mentioned above.
2) The subjects conduct exercise training at home through a remote platform without face-to-face supervision of physical therapists, and may also cause the above three risks due to non-standard movements.
3) The mental health related risks caused by the decline of sports confidence and the deterioration of mood caused by the difficulty in meeting the expectations of the subjects or their inability to regularly adhere to sports training.

5.2 For possible risks, risk prevention and treatment mechanisms have been formulated:

5.2.1 Interactive Health coaching course via video connection:

1) Physiotherapists shall formulate personalized exercise plans for subjects through professional, detailed, and personalized exercise tests, deliver correct and appropriate educational information, supervise subjects to perform adequate and reasonable warm-up activities, and implement exercise intervention according to their exercise performance and health condition. In addition, patients will be equipped with heart rate belts to monitor their exercise intensity. Prevent sports injuries and post-exercise symptoms and other risks directly related to sports.
2) When the subject has pain that prevents him from continuing to exercise, aggravation of movement restriction symptoms, dizziness and other conditions, the APP will issue an alarm and forcibly exit the exercise interface, and place the subject to rest until the symptoms are relieved. If the symptoms cannot be

relieved for more than 10 minutes, according to the clinical manifestations immediately take corresponding treatment or rescue measures.

3) The subject must conduct exercise training under the care or supervision of other family members. In case of emergency, the subject will be handled by family members or make emergency call.
4) Subjects who are not supervised by other family members during exercise training related to the project will be arranged to the nearest community medical institution for exercise training, and corresponding treatment or rescue measures will be taken immediately in accordance with the relevant professional clinical rescue treatment norms in case of falling, chest pain, sudden onset of cardiovascular and cerebrovascular diseases in the hospital environment.
5) Emergency contact numbers: 112 (First Aid), 18810218262 (Clinical Coordinator), 18991463887 (project contact).

5.2.2 Subjects' home exercise training:

1) The physical therapist will inform the subjects to carry out exercise training including warm-up, training, cooling, stretching, and other steps in strict accordance with the personalized exercise program, and make reasonable exercise progression according to their own activity tolerance, exercise performance and irritability of symptoms, and each progression should be carried out after reaching an agreement with the physical therapist. To prevent sports injury caused by improper sports and other directly related risks.
2) The subject will conduct exercise training under the care or supervision of other family members. In case of an emergency, the subject will be handled by family members or make emergency calls.
3) The subjects need to record the adverse events and pain degree through the exercise diary on the remote platform. The clinical coordinator will check the self-assessment results of the patients participating in the remote exercise training at a fixed time every day. When the patients find severe pain aggravation or record missing, the coordinator will call for an inquiry.

4) Emergency contact number: 112 (First aid), 18810218262 (Clinical coordinator), 18991463887 (project leader).

Please note:

1. You must ensure the safety and stability of the exercise training environment and exercise under the supervision and care of your family members to ensure safety, no matter whether you have a video connection with a physical therapist.
2. If you are an elderly person living alone, we will arrange for you to go to a nearby community medical institution for exercise training under the supervision of professionals.
3. You must strictly follow the physiotherapist's personalized exercise plan, which is customized according to your condition, assessment results and exercise performance. Any progression and changes to the exercise plan should be decided after discussion with the physiotherapist to prevent exercise-related injuries.
4. When sports injury occurs during sports exercise at home, you need to perform a series of emergency treatment methods such as cold compress, brake, elevation and pressure dressing for sports injury. Specific methods will be demonstrated and taught before the intervention begins.
5. If you experience symptoms such as aggravated symptoms and dizziness during exercise, you should stop training and rest for 10 minutes. If the symptoms still do not improve, you should immediately enter the medical emergency procedure.

When the above risks occur during the treatment period, adverse events caused by this study will be treated by the researcher free of charge. For serious adverse events judged to be related to clinical trials, the researcher will treat them free of charge. For the economic losses caused, the research group will provide economic compensation of corresponding value, including a transportation subsidy and accommodation subsidy of 300 yuan. The certificate of loss with legal value shall prevail.

6. What are the benefits you can take from the study?

Participating in this study, you are likely to experience improvements in function, pain relief, scientific self-management habits, and improved mental health and quality of life. This research will also help determine which treatments are safer and more effective for other patients with conditions similar to yours. But these benefits are not guaranteed.

7. Are there any fees that need to pay during the study?

Participants will not be required to pay any fees for participating in this study. For participants who complete all interventions and evaluations during the intervention period, we will provide 300 yuan of transportation allowance and accommodation allowance.

8. Is personal information confidential?

Your study data will be stored at West China Hospital of Sichuan University, and your medical records will be accessible to the investigator, the study authority, and the ethics Review Committee. Any public report of the results of this study will not disclose your personal identity. We will make every effort to protect the privacy and personal information of your personal medical data to the extent permitted by law.

9. Must I participate in a study?

Participation in the study is completely voluntary and you may refuse to participate in the study or withdraw from the study at any stage of the study without discrimination or retaliation, without affecting your medical treatment and rights. If you decide to withdraw from the study, please contact your physical therapist or program manager for proper diagnosis and treatment. Project leader Tel: 18991463887 (Yuan-Feng).

Patient Statement:

I have read the above presentation on this study, and my researchers have fully explained to me the purpose of this study, its operational process, and the possible risks and potential benefits of participating in this study and answered all my relevant questions.

I understand the purpose of this study and I am free to withdraw at any time without medical cares or legal rights being affected. I am voluntary to participate in this study.

I understand that results of my visits may be shared with the research team of West China Hospital of Sichuan University.

I agree to allow any information provided to be medical research upon the understanding that my identity will remain anonymous wherever possible.

Please indicate your wishes in the below scenarios:

Pleas tick or initial yes or no:	Please tick √ or initial	
	YES	NO
I agree for my details to be shared and used in further research that be running by West China Hospital of Sichuan University	☐	☐

Patient (to be completed by the patient):

Signature: ____

Name (block letters):

____

Date: ____

Phone:

____

Legal representative (block letters, if applicable): ______________________

Relationship with patient:

____

Witness (block letters, if applicable):

____

Date: ____

Investigator Statement:

I have explained the request to the above-named patient, particularly, the ethical principles, risks, benefits, free, voluntariness and confidentiality that may arise from

participating in this study. And he/she has indicated his/her willingness for participating in this study.

Signature: ____

Name (block letters): ____

Date: ____

Ethics Committee on Biomedical Research,

West China Hospital of Sichuan University

Tel: 028-85422654!028-85423237

(1 copy for patient; 1 held in patient notes, original stored in Investigator Site File)
