# Supplementary figures and images for: Effect of telemedicine-supported structured exercise program in patients with chronic low back pain: a randomized controlled trial
Source: PLoS One. 2025 Jun 25;20(6):e0326218. doi: 10.1371/journal.pone.0326218 (PMC12193851; doi:10.1371/journal.pone.0326218)

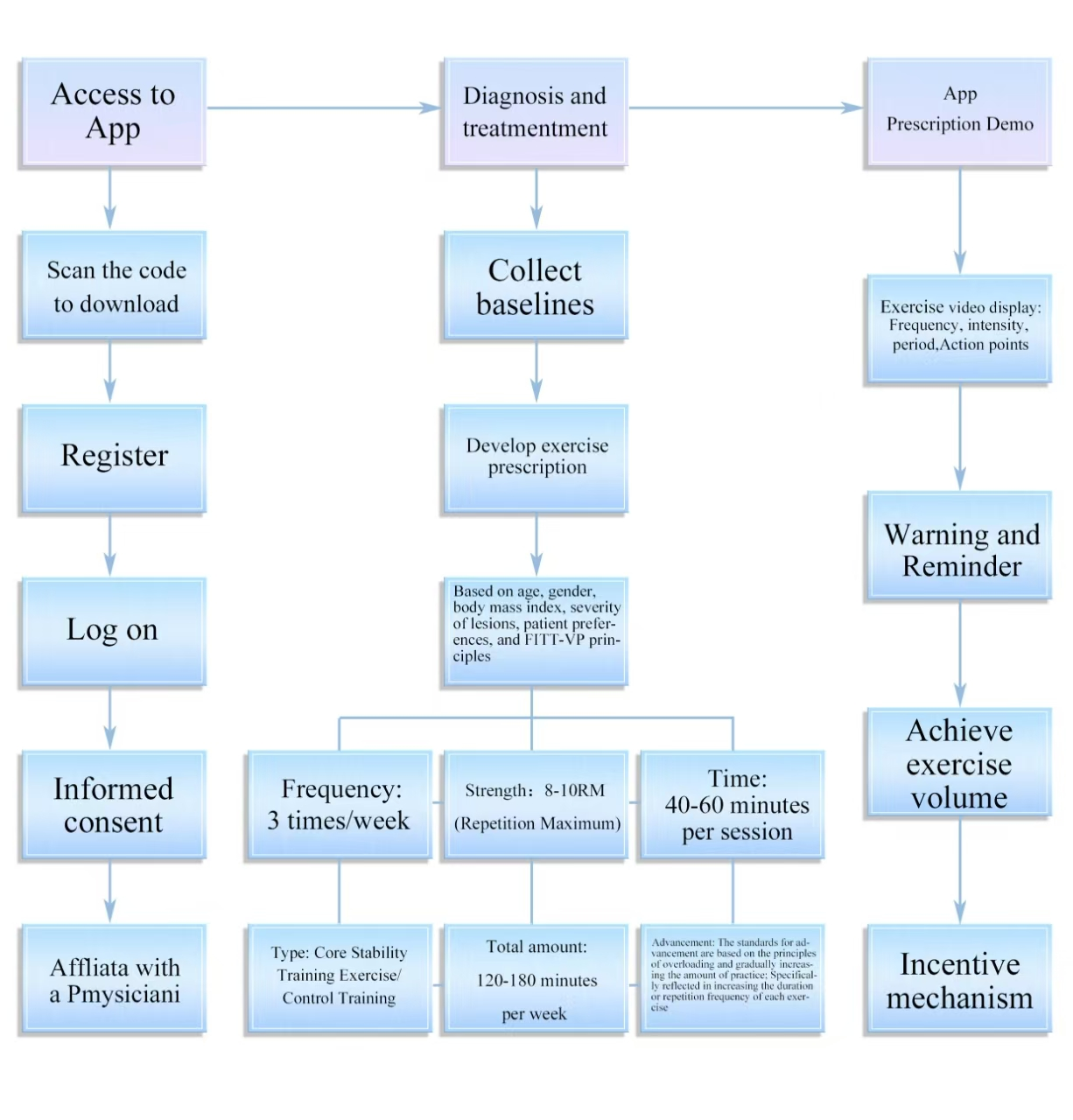

Supplement: S1 Fig — (TIF) [file pone.0326218.s004.tif]

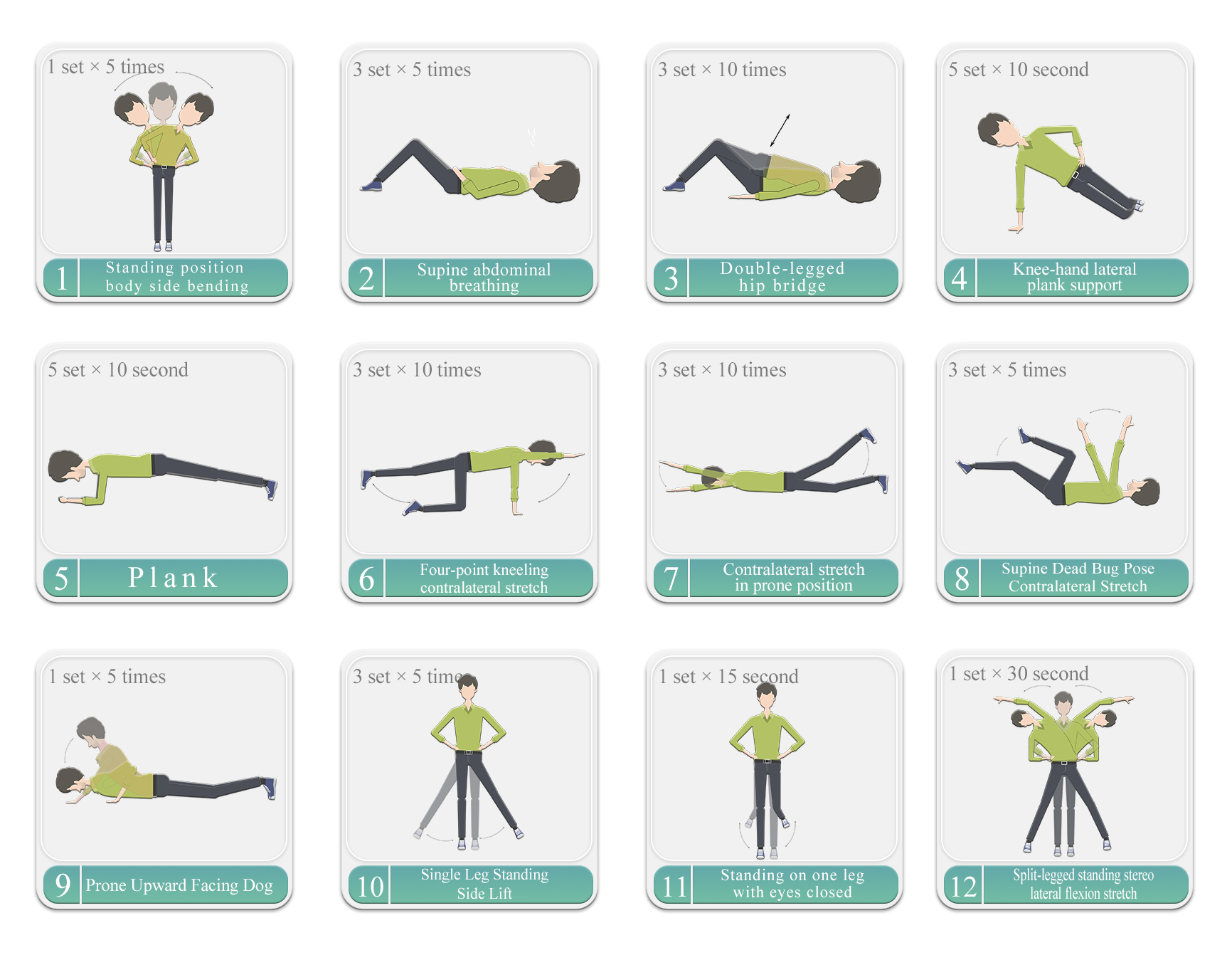

Supplement: S2 Fig — (TIF) [file pone.0326218.s005.tif]

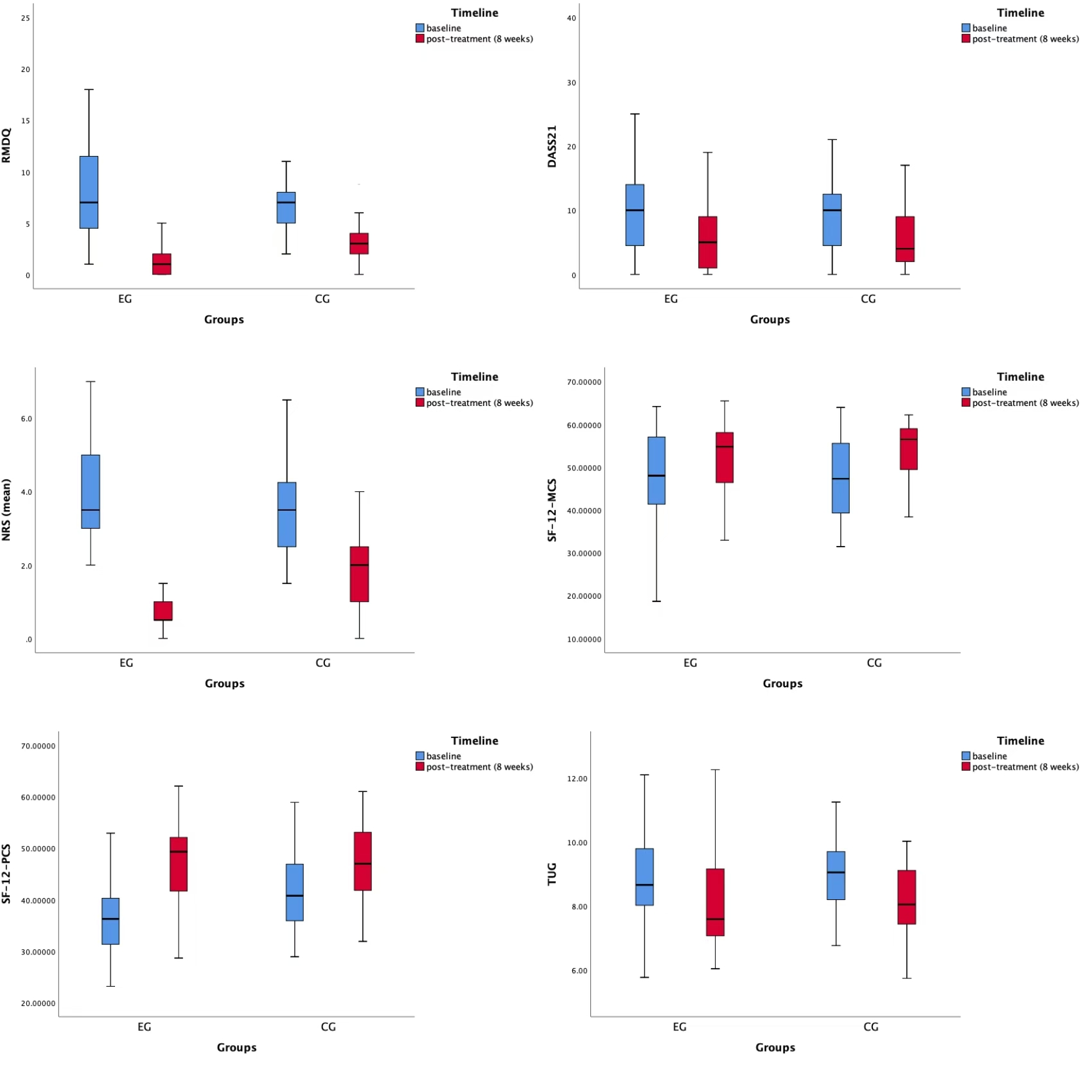

Supplement: S3 Fig — (TIF) [file pone.0326218.s006.tif]
