# Supplementary material for: Effect of telemedicine-supported structured exercise program in patients with chronic low back pain: a randomized controlled trial
Source: PLoS One. 2025 Jun 25;20(6):e0326218. doi: 10.1371/journal.pone.0326218 (PMC12193851; doi:10.1371/journal.pone.0326218)
Supplement: S1 Table — (DOCX) [file pone.0326218.s007.docx]

S1 Table. Interaction effect test for each indicator (except for the exercise adherence indicator)* PP analysis.（N=71）

| Outcome Variables | | Cases | P-value for between-group effect  (Group) | P-value for within-group effect  (Time) | P-value for interaction effect  (Group*time) |
| --- | --- | --- | --- | --- | --- |
| RMDQ | | 225 | 0.07 | **＜0.001** | **＜0.001** |
| NRS (mean value) | | 225 | **0.001** | **＜0.001** | **＜0.001** |
| DASS21 | | 225 | 0.6 | **＜0.001** | 0.48 |
| SF-12 |  | | | | |
|  | PCS | 225 | 0.18 | **＜0.001** | **0.04** |
|  | MCS | 225 | 0.77 | **＜0.001** | 0.63 |
| TUG | | 178 | 0.84 | **＜0.001** | 0.97 |

Abbreviations: PCS, physical component summary; MCS, mental component summary

^#^Linear mixed-effects model for the repeated-measures analysis using the compound symmetric covariance structure

*Linear mixed-effects model based on PP analysis
