# Supplementary material for: Effect of telemedicine-supported structured exercise program in patients with chronic low back pain: a randomized controlled trial
Source: PLoS One. 2025 Jun 25;20(6):e0326218. doi: 10.1371/journal.pone.0326218 (PMC12193851; doi:10.1371/journal.pone.0326218)
Supplement: S2 Table — (DOCX) [file pone.0326218.s008.docx]

S2 Table. Outcomes changes (except for the exercise adherence indicator)* PP analysis. (N = 71)

| Outcome Variables (median/mean) | N | EG | N | CG | Estimate value | P* |
| --- | --- | --- | --- | --- | --- | --- |
| Comparison between groups | | | | | | |
| **RMDQ** | 37 |  | 34 |  |  |  |
| Baseline |  | 7（4-12） |  | 7（5-8） | -3.85 | ＜0.001 |
| 8 weeks |  | 1（0-2） |  | 3（2-4.25） |  |  |
| **NRS** | 37 |  | 34 |  |  |  |
| Baseline |  | 3.5（3-5） |  | 3.47±1.28 | -1.86 | ＜0.001 |
| 8 weeks |  | 0.5（0.25-1） |  | 1.96±1.03 |  |  |
| **SF-12-**  **PCS** | 37 |  | 34 |  |  |  |
| Baseline |  | 37.1±7.18 |  | 41.51±7.41 | 3.87 | 0.02 |
| 8 weeks |  | 46.85±8.02 |  | 46.80±7.81 |  |  |
| Comparison within group | | | | | | |
| **DASS21** | 37 |  | 34 |  |  |  |
| Baseline |  | 10（4-15） |  | 10（4-13） | -3.78 | ＜0.001 |
| 8 weeks |  | 5（1-10） |  | 4（2-9） |  |  |
| **SF-12-**  **MCS** | 37 |  | 34 |  |  |  |
| Baseline |  | 47.78±10.31 |  | 47.18±9.14 | 4.84 | ＜0.001 |
| 8 weeks |  | 54.77（45.9-58.45） |  | 56.51（49.17-59.01） |  |  |
| **TUG** | 10 |  | 12 |  |  |  |
| Baseline |  | 8.9±1.46 |  | 9±1.12 | -0.92 | ＜0.001 |
| 8 weeks |  | 7.6（7.07-9.2） |  | 8.05±1.13 |  |  |

^#^Linear mixed-effects model for the repeated-measures analysis using the compound symmetric covariance structure

*Linear mixed-effects model based on PP analysis
