# Supplementary material for: Effect of telemedicine-supported structured exercise program in patients with chronic low back pain: a randomized controlled trial
Source: PLoS One. 2025 Jun 25;20(6):e0326218. doi: 10.1371/journal.pone.0326218 (PMC12193851; doi:10.1371/journal.pone.0326218)
Supplement: S3 Table — (DOCX) [file pone.0326218.s009.docx]

S3 Table. Comparison of EARS at each time point between the two groups *PP analysis. (N=71)

|  | T1 | T2 | Difference |
| --- | --- | --- | --- |
| EG | 49（46～51） | 48（45～56） | 0（-2.5～3） |
| CG | 43.68±3.6 | 45.35±4.89 | 1.44±4.33 |
| *Z* | -5.33 | -2.83 | 1.59 |
| *P* | **＜0.001** | **0.005** | 0.11 |

Abbreviations: T1, Last day at 4 weeks of intervention; T2, last day at 8 weeks of intervention.

*Mann-Whiteney U test
